# Single-Crystal HfB_2_ Nanorod-Induced Synergy in HfB_2_–SiC Ultrahigh-Temperature Ceramics: Enhancement of Mechanical and Ablation Resistance

**DOI:** 10.34133/research.0963

**Published:** 2025-11-06

**Authors:** Kewei Li, Zhen Wang, Mulan Yu, Mengen Hu, Zhulin Huang, Yuan Cheng, Xiaoye Hu, Yue Li, Ping Hu, Xinghong Zhang

**Affiliations:** ^1^ Key Laboratory of Materials Physics and Anhui Key Laboratory of Nanomaterials and Nanotechnology, Institute of Solid State Physics, HFIPS, Chinese Academy of Sciences, Hefei 230031, China.; ^2^ University of Science and Technology of China, Hefei 230026, China.; ^3^ Harbin Institute of Technology, Harbin 150001, China.; ^4^ Tiangong University, Tianjin 300387, China.

## Abstract

Achieving a synergistic improvement in the toughness and oxidation resistance of boride ultrahigh-temperature ceramic composites remains a challenge for advancing new-generation hypersonic vehicles. In this study, HfB_2_–SiC composites were synthesized by integrating self-made single-crystal HfB_2_ microrods with commercial powders via spark plasma sintering, which exhibited enhancement of mechanical and ablation resistance. Compared to the sample without addition, the incorporation of 6 wt.% single-crystal HfB_2_ microrods into the HfB_2_–SiC composites resulted in a 4.1% increase in hardness and a 37.6% improvement in fracture toughness, reaching 15.45 ± 0.89 GPa and 7.58 ± 0.66 MPa·m^1/2^, respectively. After static oxidation at 1,500 °C in air for 300 min, the ceramic block supplemented with 3 wt.% HfB_2_ microrods exhibited a minimal weight gain (0.018 mg/cm^3^). Upon exposure to a plasma flame at 2,000 °C for a period of 60 s, the material demonstrated a mass ablation rate of −0.013 mg/s and a linear ablation rate of 0.25 μm/s. Based on the experimental results, the excellent oxidation and ablation resistance might be related to the naturally low reactivity of the exposed $\langle 10\bar{1}0\rangle$ crystal planes present on HfB_2_ microrods, which aligns with the findings from first-principles calculations. This approach improves the comprehensive performance of the material by leveraging the inherent strengths of single-crystal HfB_2_ microrods and provides a promising design concept for HfB_2_ composites, which lays both theoretical and material groundwork for the development of a new generation of hypersonic vehicles.

## Introduction

The development of hypersonic vehicles imposes stringent requirements on thermal protection materials (TPMs) for critical components, necessitating simultaneous enhancement of mechanical properties and oxidation–ablation resistance under extreme operational conditions [1–3]. Among several candidates for TPMs, ultrahigh-temperature ceramics (UHTCs) demonstrate marked potential for application in extreme thermal protection fields (the nose cone and leading edge of the wing rudders of hypersonic aircraft, for instance) due to their excellent high-temperature stability, oxidation resistance, and ablation resistance [4,5]. However, UHTCs are hampered by a deficiency in fracture toughness, which makes it necessary to integrate with anisotropic reinforcement materials (such as whiskers, fibers, or rodlike structures) during application. In such composite material systems, enhanced mechanical properties are achieved via mechanisms such as crack deflection, bridging, fiber pullout, and load transfer [6–10]. Comparatively, employing a composite with carbon fibers is a widely used and effective approach for augmenting material toughness; the mechanical properties of ceramic composites can be promoted by the high aspect ratio, specific strength, and specific modulus of carbon fibers [6,11–13]. However, in high-temperature oxygen-rich environments, when cracks occur to the composite material, the carbon component serving as the toughening phase undergoes rapid oxidation ablation, leading to a severe deterioration in the structural mechanical properties and catastrophic structural damage [14–16]. Introducing submicron-sized SiC powders into UHTCs is another commonly used method for toughening. Through the pinning effect of SiC grains, the diffusion growth of crystals is hindered, thereby achieving grain refinement and improving the mechanical properties. Nevertheless, in this system fabricated from polycrystalline materials, both the thermal shock resistance and creep resistance are notably inferior, presenting substantial risks for its utilization at elevated temperatures [17,18]. Under such circumstances, enhancing the intrinsic toughness of ceramic materials through intrinsic nanorods or whiskers and then combining them with carbon fibers may suppress crack initiation and hinder crack propagation toward the carbon fiber reinforcements, thereby greatly improving the mechanical properties of ceramic matrix composites [19]. Furthermore, using carbon fibers as the substrate and rodlike powders and commercial powders as raw materials, in situ preparation of gradient composite materials is expected to further improve the mechanical properties [20,21]. In general, if the addition of the toughening phase can improve its oxidation resistance and ablation resistance at the same time, the synergistic improvement of mechanical resistance and ablation resistance can be achieved, improving the structural reliability of the composite material under various complex load conditions (thermomechanical–chemical coupling effect) [22,23].

To achieve the effect, the incorporated toughening phase must possess a one-dimensional anisotropic structure while exhibiting excellent oxidation and ablation resistance. Generally, single-crystal structures largely eliminate grain boundaries, thereby avoiding the preferential oxidation at grain boundaries that is commonly observed in polycrystalline materials, and thus provide superior oxidation and ablation resistance. Consequently, incorporating one-dimensional single-crystal nanowires or nanorods as toughening phases into ceramic composites can induce a toughening effect within the matrix system while simultaneously enhancing the oxidation/ablation resistance of UHTCs, expected to achieve a synergistic improvement of both mechanical properties and oxidation/ablation resistance. Nevertheless, research on this synergistic enhancement of mechanical properties and oxidation/ablation resistance remains relatively limited. The primary challenge lies in the fabrication of one-dimensional single-crystal UHTCs powders with uniform structures [24–29], which restricts their application in UHTC-based composites. This difficulty has also led to the absence of research on the relationship between the microstructure of UHTCs composites and their oxidation and ablation properties.

Motivated by this theoretical framework and existing challenges, we developed a multiscale hierarchical architecture through the integration of self-synthesized multibranched single-crystal HfB_2_ nanorods as an anisotropic toughening phase. Cascade optimization using single-crystal HfB_2_ nanorods and commercial powders, followed by spark plasma sintering (SPS), was employed to fabricate HfB_2_–SiC composites with increased mechanical robustness and ablation resistance. In contrast to the conventional HfB_2_–SiC composite, the incorporation of a rodlike morphology is anticipated to strengthen the fracture toughness of the material via crack deflection, bridging, and other mechanisms. Compared with samples without added toughening phases, the hardness and fracture toughness of the ceramic bulk with 6 wt.% HfB_2_ microrods added have respectively increased by 4.1% and 37.6%. First-principles calculations indicate that for HfB_2_ crystals, their (0001) planes are more prone to contacting with the external environment and thus react more readily compared to the $\langle 10\bar{1}0\rangle$ planes, whereas for single-crystal HfB_2_ microrods, the $\langle 10\bar{1}0\rangle$ planes are more exposed, which might help to improve the material’s oxidation and ablation resistance. Combined with a plasma flame ablation experiment, it is verified that the composite with 3 wt.% HfB_2_ microrods has excellent ablative properties, with a mass ablation rate of −0.013 mg/s and a linear ablation rate of 0.25 μm/s. These results demonstrate the dual role of HfB_2_ microrods in improving mechanical properties and oxidation/ablation resistance. Compared with other microrod- or fiber-reinforced UHTC systems, the single-crystal HfB_2_ microrods in this work have the following advantages: Firstly, the microrods have the same matrix as the base material, so there is no thermal mismatch between the reinforced phase and the matrix. Secondly, the advantages of the single crystal can be fully utilized in this composite material system. The hexagonal HfB_2_ has higher hardness in the direction of the *a* axis, which is beneficial to improving the mechanical properties of the material. In addition, the high thermal conductivity in the direction of the *c* axis will accelerate the thermal diffusion rate at the surface of the sample during ablation, reducing the surface temperature of the material, thus improving the ablation resistance of the material.

## Results and Discussion

### Multiscale microstructure characterization

**Fig. 1.**
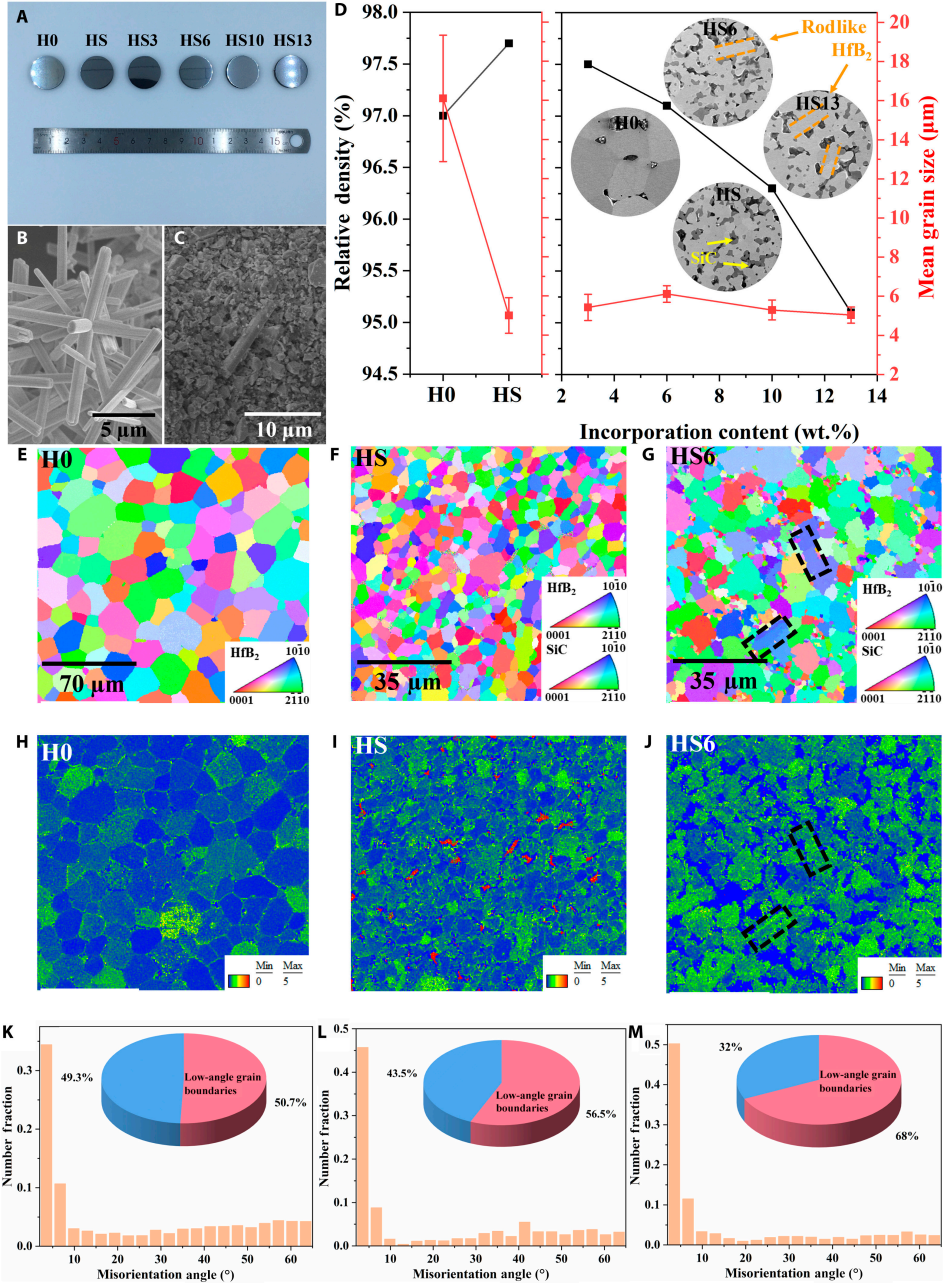
Macrographic image of the ceramic bulks after spark plasma sintering (SPS) (A), coupled with scanning electron microscopy (SEM) images of the multibranched HfB_2_ powders (B) and pre-compact green body with 6 wt.% HfB_2_ microrod incorporation (C). The impact of the addition of HfB_2_ microrods on the relative density and grain size (D) along with SEM microstructures of the polished surface ceramic bulks (inset of (D)). Crystallographic orientation (CO) maps (E to G), kernel average misorientation (KAM) maps (H to J), and misorientation angle distributions (MADs) (K to M) of HfB_2_ and SiC grains in H0, HS, and HS6 ceramic bulks (the black rectangular area is where the single-crystal HfB_2_ microrod is located).

Figure 1A presents the optical imagery of polished ceramic blocks after SPS, each with a diameter of 20 mm and denoted as H0, HS, HS3, HS6, HS10, and HS13, based on their compositional constituents. Among them, H0 is a ceramic bulk synthesized from commercial HfB_2_ powders, while HS is a bulk prepared by adding 20 vol.% SiC to commercial powders, both of which serve as control samples for this study. HS3, HS6, HS10, and HS13 ceramic bulks were synthesized by incorporating 3, 6, 10, and 13 wt.% single-crystal HfB_2_ microrods, respectively, into the base formulation HS. Figure 1B and C present the scanning electron microscopy (SEM) images of multibranched HfB_2_ microrods and typical mixed powders (with 6 wt.% HfB_2_ microrod incorporation). The HfB_2_ microrods demonstrate a high aspect ratio and maintain a consistent morphology. In a previous work, the test results indicated that each of its branches was a highly crystalline single crystal [29]. After adequate ultrasonication and stirring, the powder mixture achieved remarkable uniformity, thereby laying the groundwork for the homogeneity of the subsequent ceramic bulk. The results presented in Fig. 1D indicate that SiC addition obviously reduces the grain size of HfB_2_ and increases the density of ceramic blocks. The pure-phase HfB_2_ sample distinctly exhibits grain growth with an average grain size of 16 μm. However, the addition of SiC results in a decrease in the average grain size to 5 μm. As the quantity of HfB_2_ microrod addition increases, there is a corresponding decrease in the ceramic body’s density, an outcome that aligns with anticipated results. Nevertheless, even when the addition reaches 13 wt.%, the density of the ceramic block consistently remains above 95%. Furthermore, the SEM images in Fig. 1D and Fig. S1 illustrate that SiC and HfB_2_ microrods are uniformly dispersed within the ceramic matrix. This distribution ensures that the samples exhibit exceptional and consistent mechanical properties [30,31].

To further investigate the interfacial bonding of single-crystal HfB_2_ microrods after being embedded into the matrix, the fracture surface morphology of the HS3 sample was analyzed in detail (Fig. S2a). Magnifying the area (Fig. S2b and c) reveals a clearer view of the grain morphology and interfacial phenomena. The fracture surface of the HfB_2_ microrods is smoother compared to commercial HfB_2_ powders, which show a distinct cleavage fracture (yellow rectangular area in Fig. S2b and c). This is due to the inherent brittleness of HfB_2_ ceramics. When the ceramic bulk fractures, the HfB_2_ microrods do not pull out directly, like carbon fibers, but rather tend to dissipate energy via transgranular fracture, thereby enhancing the fracture toughness of the material. Near the grain boundary of the HfB_2_ microrods (pink marks in Fig. S2a and b), one can discern closely packed SiC grains (gray phase) and HfB_2_ grains (white phase), with no obvious pores. The energy-dispersive spectroscopy (EDS) mapping of the cross-section reveals a relatively uniform distribution of various elements.

Due to crystallographic orientation (CO), kernel average misorientation (KAM) and misorientation angle distributions can greatly affect the mechanical properties of the material; electron backscatter diffraction characterization of H0, HS, and HS6 ceramic bulks was conducted as shown in Fig. 1E to G. It is obvious from the CO maps that no texture was detected in these samples. The HS6 sample revealed the presence of single-crystal HfB_2_ microrods (black rectangular area in Fig. 1G), which manifest as a singular grain orientation in the CO map (the $\left(10\bar{1}0\right)$ direction). Figure 1H to J display the KAM maps of H0, HS, and HS6. It can be concluded that the KAM value of the HS bulk in certain regions is evidently elevated compared to that of the pure-phase HfB_2_ bulk and the sample with 6 wt.% HfB_2_ microrods added. This suggests that the material possesses regions of stress concentration, which could potentially result in its cracking and fragmentation under high-temperature conditions. Furthermore, the grain boundary angle distribution illustrated in Fig. 1K to M demonstrates that the H0 ceramic bulk contains the lowest proportion of low-angle grain boundaries (<15°), while the HS6 bulk exhibits the highest. This phenomenon can be ascribed to the incorporation of HfB_2_ microrods and SiC powders, which effectively suppress grain boundary migration and growth. Generally, the growth of grains is mainly achieved through grain boundary diffusion. Low-angle grain boundaries grow through diffusion to form high-angle grain boundaries; a higher concentration of low-angle grain boundaries suggests enhanced mechanical properties of the material [32,33]. The integration of single-crystal HfB_2_ microrods can improve the mechanical attributes of the material by modifying both the grain boundary angle distribution and the dislocation density distribution.

### Mechanical properties

To analyze the cross-scale (micro–macro) and multiparameter (hardness, modulus, and fracture toughness) characteristics of HfB_2_–SiC materials and elucidate the reinforcing role of single-crystal HfB_2_ microrods in the composite system in detail, the Vickers hardness and microhardness of these composites were tested separately.

#### Vickers hardness

**Fig. 2.**
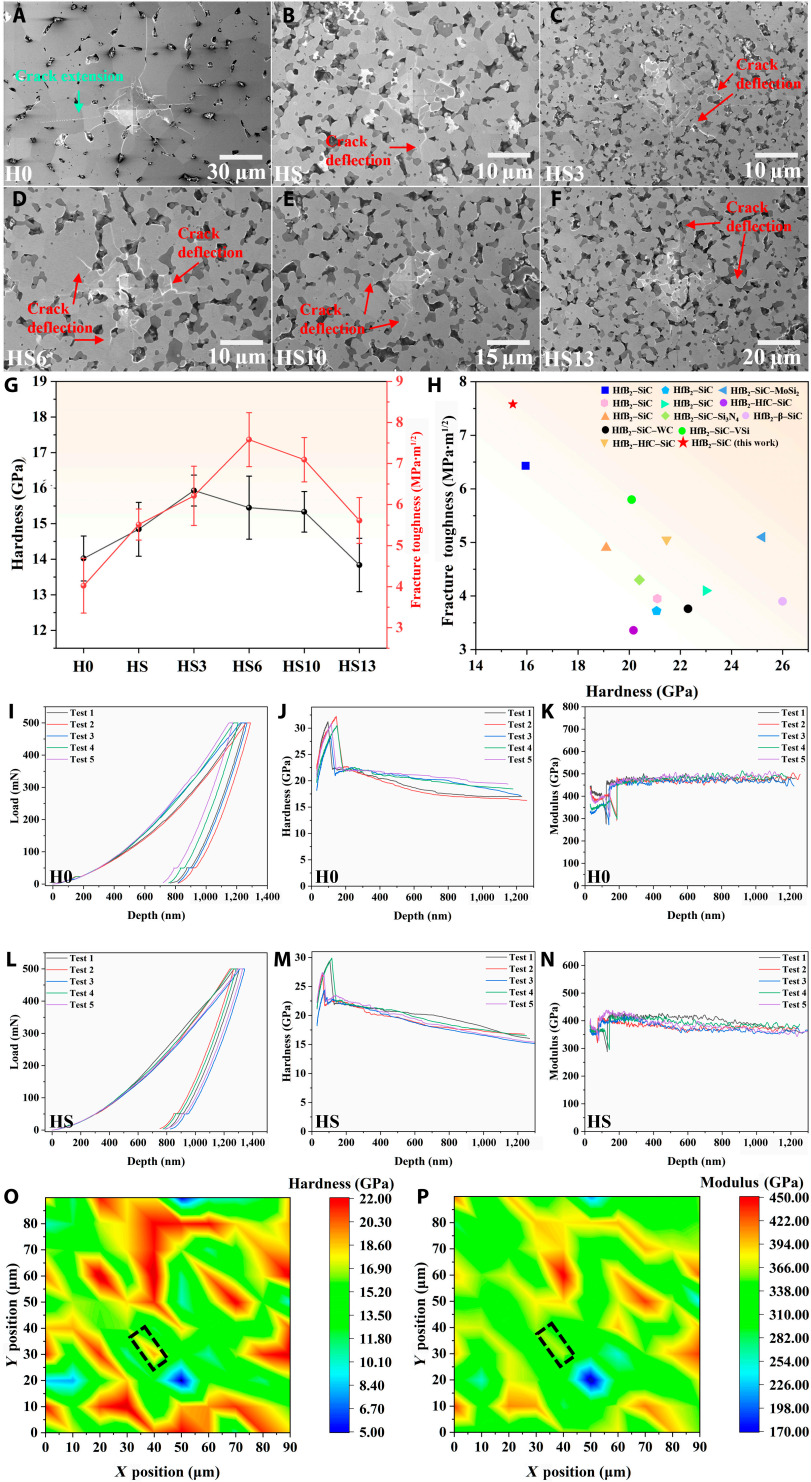
The microscopic morphologies of the indentations on the polished surfaces of H0 to HS13 ceramic bulks (A to F). The impact of HfB_2_ microrod incorporation on the hardness and fracture toughness of H0 to HS13 (G), as well as comparison with the properties of HfB_2_-based ceramics reported in the literature (H). Load–displacement, hardness–displacement, and Young’s modulus–displacement curves of H0 (I to K) and HS (L to N) bulks and the hardness (O) and modulus (P) mapping in the HS6 bulk (the black rectangular area is where the single-crystal HfB_2_ microrod is located).

Figure 2A to F depict the indentation morphology of each sample under a force of 4.9 N. It is evident that the H0 bulk, which lacks any toughening phase, exhibits an undeflected crack extension of HfB_2_ ceramic. Upon the integration of SiC powders, HfB_2_–SiC ceramics exhibit minor crack bending, as depicted in Fig. 2B. The presence of SiC grains induced a degree of crack deflection and energy dissipation throughout the crack growth process. On this basis, after the addition of HfB_2_ microrods, examination of the microscopic morphology of indentation (Fig. 2C to F) reveals that crack lengths have been reduced, and the trajectories of these cracks exhibit greater complexity. The intricate nature of cracks can effectively dissipate the energy associated with crack propagation, thereby enhancing the fracture toughness of the material [34,35]. The HS6 ceramic bulk with the highest fracture toughness was selected to compare the crack propagation on the surfaces of H0 and HS ceramic bulks. In SEM images, cracks on the H0 bulk’s surface extend almost linearly; it is a typical transgranular fracture (Fig. S3a to c). When SiC powders are added to the ceramic bulk, the grain size notably decreases, leading to a more intricate crack propagation path on the surface, characterized by the coexistence of both transgranular and intergranular fractures (Fig. S3d to f). This dual-fracture mode causes the crack to deflect during propagation, effectively dissipating energy and improving fracture toughness. Upon the addition of single-crystal HfB_2_ microrods, the material maintains a relatively small grain size. As the crack navigates through the HfB_2_ microrod, it bridges and deflects (Fig. S3g and h), further complicating its path on the ceramic block surface. This results in increased energy dissipation, thereby boosting the material’s fracture toughness.

The mechanical properties of H0 to HS13 ceramic bulks are elucidated in Fig. 2G. The data reveal that the Vickers hardness of HS ceramics under such test conditions is 14.84 ± 0.76 GPa, while the fracture toughness is 5.51 ± 0.38 MPa·m^1/2^. Upon the addition of 3 wt.% HfB_2_ microrods, there is an increase in the Vickers hardness of the ceramics, reaching 15.93 ± 2.43 GPa, which is the highest among several samples. Subsequently, when the quantity of HfB_2_ microrods is augmented to 6 wt.%, the fracture toughness of the ceramic block is further improved, reaching 7.58 ± 0.66 MPa·m^1/2^, which is increased by 37.6% compared with that of the HS bulk. The hardness of the HS6 bulk is slightly lower than that of the HS3 bulk, which is 15.45 ± 0.89 GPa, but is still increased by 4.1% compared with that of the HS bulk. Weibull distribution diagrams are made based on the data and show that all the data are within the confidence interval (Fig. S4a and b). Compared to the mechanical properties of HfB_2_-based ceramics as described in previous literature [30,31,36–44], the HfB_2_–SiC composites evaluated in this study exhibit lower hardness, yet superior fracture toughness (the data are shown in Table S1). This discrepancy can likely be attributed to variations in the force applied during the testing process. Nevertheless, a longitudinal comparison of the work test data reveals that HfB_2_–SiC composites with single-crystal HfB_2_ microrods exhibit exceptional Vickers hardness and fracture toughness.

#### Microhardness hardness

Nanoindentation tests were performed on the polished surfaces of the H0, HS, and HS6 bulks. Figure 2I to K depict the load–displacement, hardness–displacement, and Young’s modulus–displacement curves of the indents, respectively. To ensure the reliability of the results, 5 distinct points on each sample were examined. According to the test results, the mean Vickers hardness of HfB_2_ grains in the H0 bulk is 20.61 GPa, while the elastic modulus is 476.57 GPa. For the HS bulk, the mean Vickers hardness of HfB_2_ grains is 20.29 GPa, and the elastic modulus is 394.05 GPa. The slight decrease in the hardness test result of the HS bulk, compared to that of the H0 bulk, can be attributed to the indentation size effect (ISE) [45]. Under identical load conditions, the H0 bulk exhibits a maximum indentation depth of approximately 1,230 nm, which is marginally less than the HS bulk’s maximum indentation depth of 1,305 nm. This reduction in indentation depth results in a higher hardness measurement value for the H0 bulk. For the HS6 bulk, due to the inability to precisely determine the position of the HfB_2_ microrods, mapping tests of hardness and Young’s modulus within a 90 × 90 μm range were conducted. The region delineated by the black rectangle in Fig. 2O and P is the area where the HfB_2_ microrod is located. Compared to the surrounding HfB_2_ particles, the hardness and modulus values within this region exhibit a slight elevation. This observation aligns with the values reported through first-principles calculations, thereby providing indirect evidence for the existence of single-crystal HfB_2_ microrods [46]. The higher hardness of HfB_2_ crystals in the *c*-axis direction also provides a foundational basis for augmenting the mechanical properties of ceramic materials.

#### Compressive stress and elastic modulus

The compressive stress for each sample is shown in Fig. S5, and the calculated elastic modulus of the ceramic bulks is presented in Table S2. The elastic modulus of the material first increases and then decreases, which is consistent with the change rule of the hardness of the material. Specifically, the elastic modulus of the H0 ceramic bulk is 562.31 GPa. When SiC powders are added, the elastic modulus increases to 685.46 GPa. When a further 3 wt.% HfB_2_ microrods are added, the maximum elastic modulus of the ceramic bulk is obtained, which is 787.64 GPa. This indicates that the HS3 ceramic bulk has the best stiffness and is not easily deformed under compressive stress.

Furthermore, the influence of the addition of single-crystal HfB_2_ microrods on the oxidation resistance of ceramic materials was also explored. Both nonisothermal and isothermal oxidation resistance were tested for samples HS3 to HS13 as a comparative measure. Additionally, H0 and HS ceramic bulks were also evaluated under identical conditions.

#### Nonisothermal oxidation

**Fig. 3.**
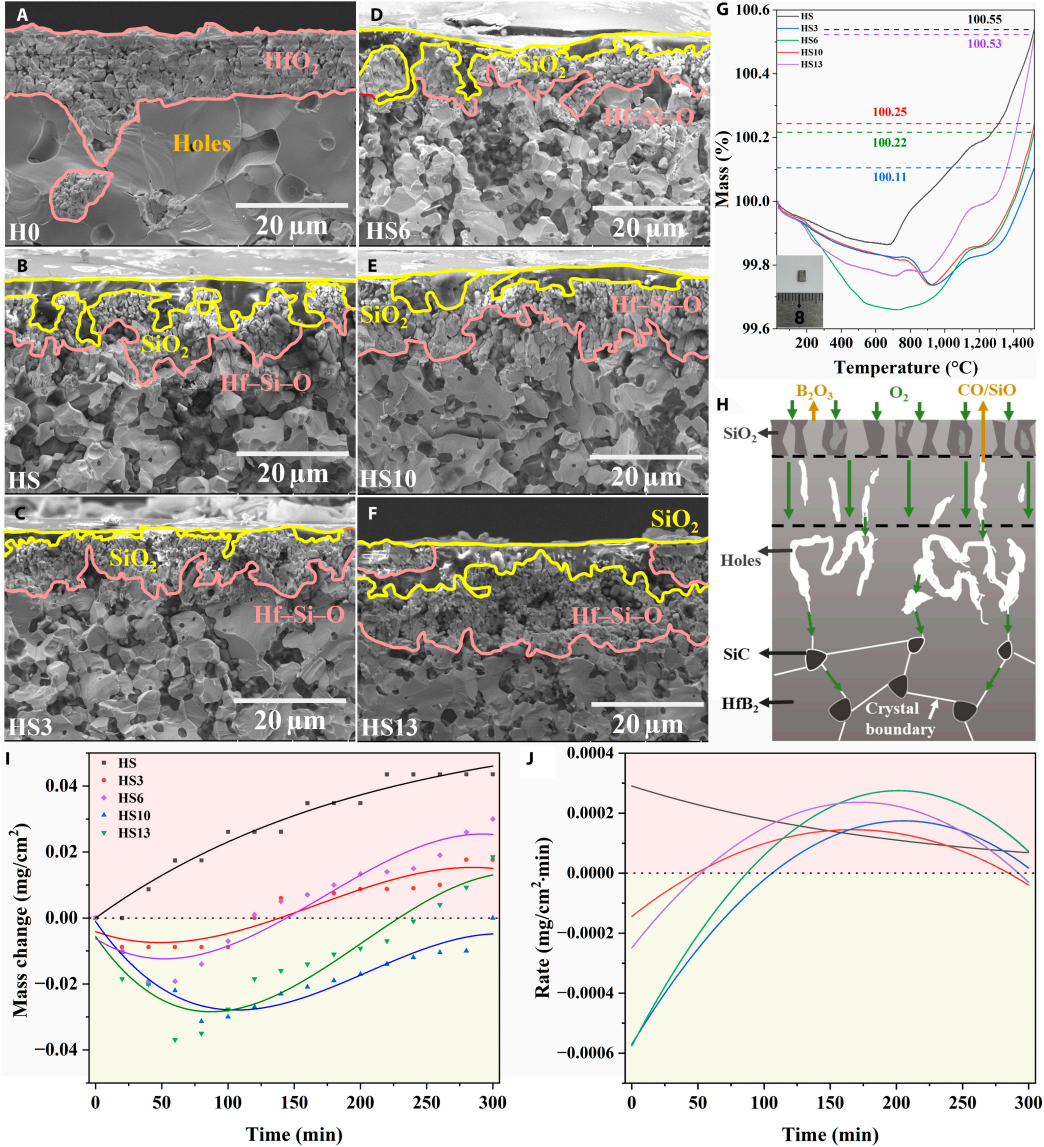
The cross-sectional oxidation morphologies (A to F) of H0 to HS13 ceramic bulks. Mass change curves of HS to HS13 ceramic bulks from room temperature to 1,500 °C in air (G) and the schematic diagram of oxidation process simulation (H). Isothermal oxidation curves of HS to HS13 ceramic bulks at 1,500 °C for 300 min in air (I and J).

Nonisothermal oxidation experiments were performed in thermogravimetric analysis–differential scanning calorimetry equipment. Figure 3A to F are the cross-sectional oxidation morphologies of the ceramic bulks after oxidation at 1,500 °C in air. It can be observed that all ceramic materials maintain a largely intact structure devoid of conspicuous cracks. However, the pure HfB_2_ bulk exhibits porosity, whereas the HfB_2_–SiC bulk is comparatively dense. This suggests the robust stability of the oxide layer at 1,500 °C. Upon integrating the EDS analysis presented in Fig. S1, the regions delineated by the yellow and red lines in Fig. 3A to F correspond to the enrichment zones of Si–O and Hf–Si–O phases, respectively. From the cross-sectional images, the oxygen-rich layer of the H0 bulk exhibits a thickness of approximately 20 μm, while the thickness of the oxygen-rich layer for the HS bulk is reduced to 6 μm. A clear conclusion can be drawn that the incorporation of SiC substantially mitigates the depth of O intrusion. The incorporation of a suitable quantity of HfB_2_ microrods can further decrease the thickness of the oxide layer. This reduction is evident in the HS3 bulk, where the oxide layer thickness has been decreased to 4 μm. EDS mapping suggests that the surface Hf–Si–O distribution of the HS3 bulk is smoother and more homogeneous than that of the HS sample. This leads to a reduced intrusion of oxygen into its interior. As the amount of HfB_2_ microrods added increases, the compactness of the system decreases, allowing oxygen to penetrate more easily during the oxidation process, leading to more severe oxidative erosion of the system. The Hf–Si–O phase, characterized by its low oxygen permeability and high-temperature stability, guarantees the antioxidation capacity of HfB_2_–SiC materials [47,48].

Figure 3G represents the mass change diagram of the ceramic material throughout the oxidation process, except for the H0 sample (Fig. S7; the H0 ceramic bulk illustrates a weight gain of approximately 5%). The oxidation process, as defined by the mass change curve, can be divided into 3 distinct stages. The initial stage extends from room temperature to approximately 700 °C. Within this phase, any residual C or B_2_O_3_ substances present within the material undergo oxidation and volatilization, resulting in a reduction of the overall material mass. Subsequently, the oxidation process transitions into its second phase, characterized by the partial oxidation of HfB_2_. Concurrently, there is an accelerated loss rate of previously generated B_2_O_3_. This dynamic results in a discernibly slower overall mass growth rate within the system. Upon reaching a temperature of 1,300 °C, SiC powders begin to undergo oxidation. This process intensifies the overall oxidation of the system and subsequently leads to an increased oxidation rate [44,49]. An examination of the entire oxidation process reveals the simulation schematic depicted in Fig. 3H; oxidation initiates at the grain boundary. As oxygen penetrates, the boundary undergoes oxidation, accompanied by the formation of oxide products and gas precipitation [50]. This process creates internal voids within the material, which in turn accelerates further oxidation. As the oxidation reaction gradually completes, the system mass reaches a relatively stable state.

#### Isothermal oxidation

To further assess the oxidation resistance of ceramic materials at 1,500 °C, isothermal oxidation experiments were conducted on ceramic bulks. As illustrated in Fig. 3I and J, after 300 min of oxidation treatment, except for the HS bulk, which demonstrates a consistent increase in mass, the weights of HS3 to HS13 bulks exhibit a pattern of initial decrease followed by an increase. The mass change per unit area of samples infused with HfB_2_ microrods remains consistently below 0.030 mg/cm^3^. Among them, the HS3 bulk exhibits a weight increase of 0.018 mg/cm^3^, which is lower than the 0.044 mg/cm^3^ observed in the HS bulk, indicating good oxidation resistance. As illustrated in Fig. 3J, during the static oxidation experiment, the oxidation rates of H3 to HS13 bulks initially display negative values. As oxidation progresses, the rate of oxidation gradually transitions from negative to positive, with the value incrementally increasing until it reaches its peak. Upon completion of the oxidation reaction, the oxidation rate reduces to nearly 0, and the quality of each system begins to stabilize. The data indicate a correlation between the rate of oxidation change in the initial phase and the relative density of the material. In materials with a lower relative density, pores can hasten the depletion of B_2_O_3_. Following this depletion, the sample experiences a rapid gain in oxidation weight. Taking HS3 and HS13 bulks as examples, the HS13 bulk exhibits a greater mass loss during the initial 75 min of oxidation. Its oxidation weight increases rapidly in the subsequent oxidation process. Upon completion of isothermal oxidation, its mass gain is nearly equivalent to that of HS3 bulk.

### Surface stability

A comprehensive examination of the HfB_2_ oxidation process at the atomic level is vital for understanding its initial oxidation mechanism. Identifying the most favorable adsorption sites for oxygen is essential prior to investigating its adsorption and diffusion behavior on HfB_2_ surfaces. The surface energy (*γ*) is an important energetic quantity used to assess surface stability. It can be calculated as

$$\gamma_{surf}=\frac{1}{2A}\left(E_{slab}-\sum_{i}N_{i}\mu_{i}\right)$$ (1)

defining *E_slab_* as the total energy of the HfB_2_ slab. *N_i_* is the number of *i* atoms, and *μ_i_* is their chemical potential. The surface energy of the stoichiometric HfB_2_ slab $\left(11\bar{2}0\right)$ (Hf:B = 1:2) is computed as

$$\gamma_{\text{surf}}=\left(E_{\text{slab}}-{\mathrm{nE}}_{\text{bulk}}\right)/2A_{s}$$ (2)

The formula for the total energy of the $\left(11\bar{2}0\right)$ slab, denoted as *E_slab_*, is given by the sum of the energy of the HfB_2_ primitive cell *E_bulk_* multiplied by the total number of primitive cells in the slab (*n*), divided by the area of the HfB_2_ surface (*A*), multiplied by 2 (2 identical surfaces). For the nonstoichiometric (0001) and $\left(10\bar{1}0\right)$ slabs, where Hf:B ≠ 1:2, surface energy can be rewritten as [51,52] (the formula derivation process is detailed in Supplementary Materials Part S2)

$$\gamma =\frac{1}{2A}\left[E_{{\mathrm{HfB}}_{2}}^{\text{slab}}-N_{\mathrm{Hf}}\times \mu_{{\mathrm{HfB}}_{2}}^{\text{bulk}}+\left(2N_{\mathrm{Hf}}-M_{B}\right)\times \left(\mu_{B}^{\text{bulk}}+\left(\mu_{B}^{\text{slab}}-\mu_{B}^{\text{bulk}}\right)\right)\right]$$ (3)

**Fig. 4.**
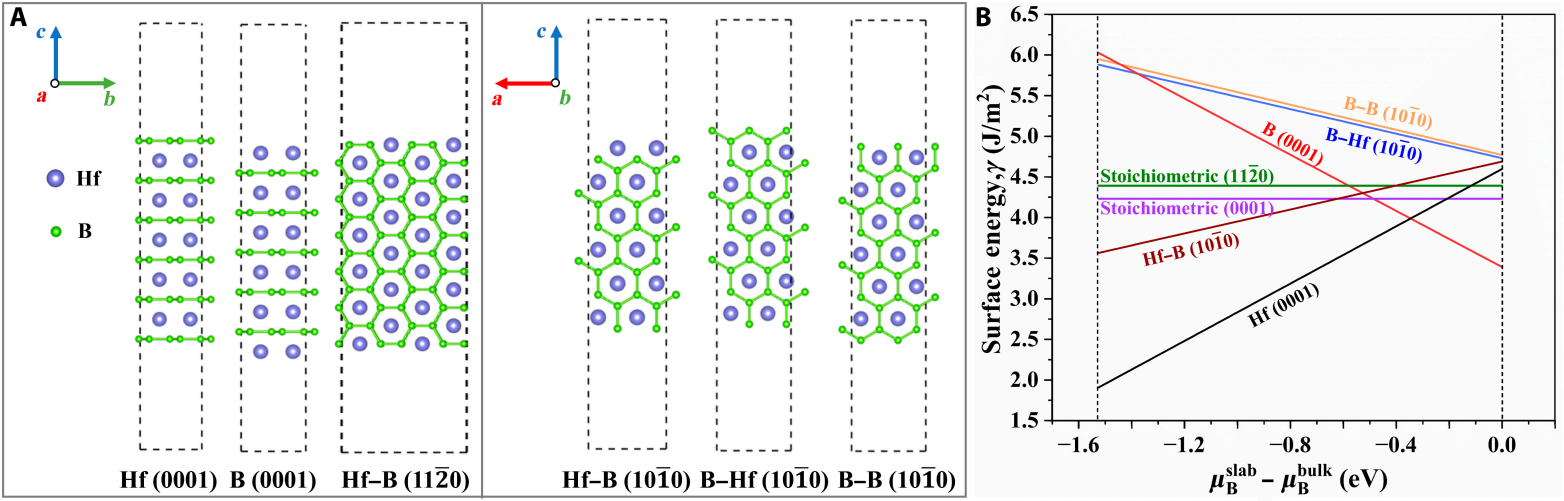
Side view of the 3 × 3 HfB_2_ slab models: Stoichiometric surfaces: Hf–B $\left(11\bar{2}0\right)$. Nonstoichiometric surfaces: Hf (0001), B (0001), Hf–B $\left(10\bar{1}0\right)$, B–Hf $\left(10\bar{1}0\right)$, and B–B $\left(10\bar{1}0\right)$ slabs (A). The relationship between the surface energy of different slabs and chemical potential (B).

The surface energies of the nonstoichiometric HfB_2_ slab exhibit a strong correlation with $\mu_{B}^{\text{slab}}-\mu_{B}^{\text{bulk}}$; that is, it is related to the formation enthalpy (*∆H*) of HfB_2_. Based on our computed formation enthalpy, the surface energies of anisotropic HfB_2_ surfaces with various terminations were determined (Fig. 4). For nonstoichiometric HfB_2_ slabs, the surface energy varies monotonically (either increasing or decreasing) with increasing $\mu_{B}^{\text{slab}}-\mu_{B}^{\text{bulk}}$. In contrast, the surface energy of the stoichiometric HfB_2_ slab remains constant. The surface energy of the nonstoichiometric structure is greatly influenced by the termination atom and surface orientation of HfB_2_. In most chemical potentials, the Hf (0001) plane exhibits the lowest surface energy, indicating superior stability compared to those of other planes. Therefore, it is more readily exposed to the outside. For single-crystal HfB_2_ microrods, their (0001) planes are located at both ends, in a state of minimal exposure.

Aizawa et al. utilized high-resolution electron energy loss spectroscopy to investigate the structure and oxidation properties of ZrB_2_. Their findings indicated that a pristine ZrB_2_ (0001) surface is terminated by the Zr layer. This Zr-terminated (0001) surface exhibits metallic characteristics and actively adsorbs gases [53–55]. Combining the CO map images in Fig. S8a and Fig. 1G, the single-crystal HfB_2_ microrods tend to be horizontally oriented within the material; that is, the $\langle 10\bar{1}0\rangle$ crystal planes are more inclined to be parallel to the sample surface, while the (0001) plane tends to be perpendicular to the sample surface. This experiment indirectly suggests that the distribution of HfB_2_ microrods within the ceramic blocks utilized in this study also exhibits similarity. Consequently, oxygen atoms, penetrating from the sample surface, initially encounter the $\langle 10\bar{1}0\rangle$ crystal planes. Due to their low reactivity, when the temperature is not high enough, oxygen atoms have to bypass these planes and adsorb on the more active Hf (0001) plane. The bypassing of oxygen atoms hinders their diffusion, slowing down the material’s internal oxidation reaction and potentially enhancing its oxidation resistance [56].

### Ablation behaviors

**Fig. 5.**
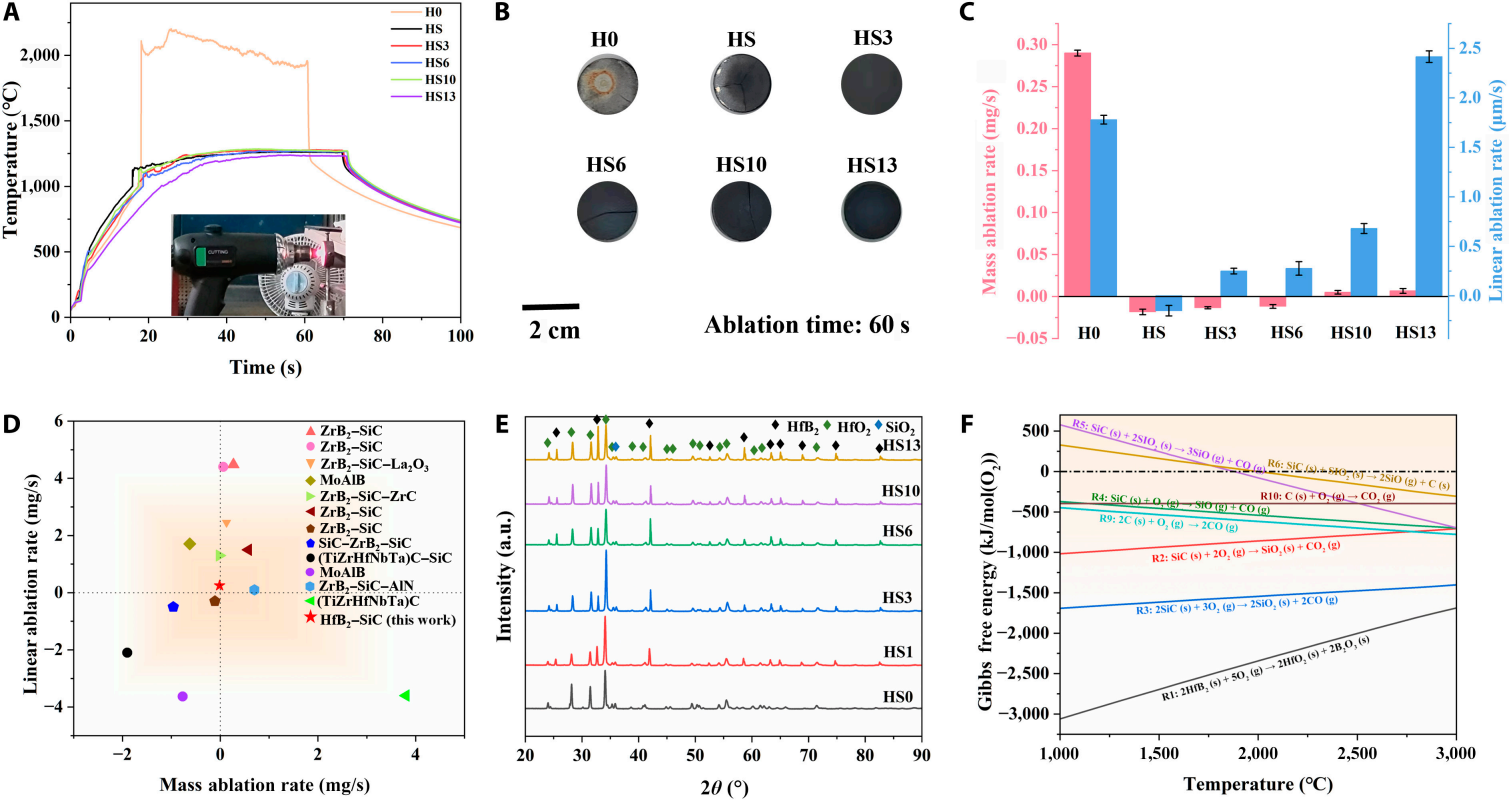
Surface temperature curves of H0 to HS13 ceramic bulks during ablation processes (A). Macrographs of the bulks after ablation (B). Mass ablation rates and linear ablation rates of the ceramic bulks after ablation for 60 s (C). Ablation-resistant performance of HfB_2_–SiC composites examined in this work compared with those of other ultrahigh-temperature ceramics (UHTCs) as reported in the existing literature (D). X-ray diffraction (XRD) patterns of ceramic bulks after ablation (E) and Gibbs free energy diagram of the oxidation reaction at different temperatures calculated by the HSC software (F).

To investigate the ablation resistance of ceramic blocks, experiments were conducted at 2,000 °C for a duration of 60 s; the inset in Fig. 5A depicts the ablation experimental process photo. The variation in surface temperature for each sample throughout the experiment is depicted in Fig. 5A. It is evident that the surface temperature of the pure-phase HfB_2_ bulk quickly escalates to a quite high temperature of approximately 2,200 °C; this phenomenon can potentially be ascribed to the comparatively inferior thermal conductivity in commercial HfB_2_ powders. In contrast, the surface temperature of the sample with 20 vol.% SiC added is reduced to 1,260 °C, which is due to the volatilization of SiO_2_ produced by SiC after oxidation, taking away a lot of heat. It indicates that the incorporation of a specific proportion of single-crystal HfB_2_ microrods into the HfB_2_–SiC system leads to a further reduction in the surface temperature of the sample during the ablation process. Upon achieving a doping level of 13 wt.% for HfB_2_ microrods, the surface temperature of the sample reaches its minimum, registering at 1,230 °C. This could be attributed to the superior thermal conductivity of the single-crystal HfB_2_ microrods in the *c*-axis direction, which further accelerates heat diffusion during the ablation process. The macroscopic surface morphology of the ceramic block post-ablation for 60 s is depicted in Fig. 5B. The image indicates that distinct ablation pits have emerged on the surface of the H0 bulk. This assertion is further substantiated by the data depicting mass and line ablation rates in Fig. 5C. The sample with 20 vol.% SiC demonstrates a notably low mass ablation rate and line ablation rate at the conclusion of the ablation process. However, the sample exhibited cracking, which may be attributed to the thermal stress experienced internally during the ablation process. In addition to this, the HS6 and HS10 bulks also exhibit varying degrees of cracking. It is imperative to mention that in the post-ablation experiment, only the HS3 and HS13 bulks preserve their original form, with their integrity remaining unaffected.

Figure 5C presents the mass and line ablation rates of different samples. The results demonstrate that among all the examined samples, HS bulk displays the most minimal mass ablation rate (−0.018 mg/s) and line ablation rate (−0.15 μm/s). Unfortunately, the integrity of the sample is compromised upon completion of the ablation, suggesting that the ablation resistance cannot be adequately assessed using only the indicators of mass ablation rate and line ablation rate. Overall, the addition of 3 wt.% HfB_2_ microrods into the sample results in enhanced ablation resistance, with a mass ablation rate of −0.013 mg/s and a line ablation rate of 0.25 μm/s. Figure 5D presents a comparative study on the line and mass ablation rates of HfB_2_–SiC composites in relation to those of other UHTCs at comparable ablation temperatures [57–66] (the data are shown in Table S3). On the whole, the HS3 ceramic bulk demonstrates exceptional ablation resistance.

The analysis of the x-ray diffraction (XRD) conducted on the surface of each sample post-ablation is depicted in Fig. 5E. The H0 ceramic block, a pure-phase HfB_2_, experienced obvious oxidation during the experiment. Consequently, the primary constituent identified in its surface is HfO_2_. The HS to HS13 bulks, enriched with SiC powders and HfB_2_ microrods, experienced lower surface temperatures during the ablation process. As a result, their surfaces do not undergo severe oxidation. The surface diffraction feature peaks are similar, mainly including the HfO_2_ phase, a small amount of the SiO_2_ phase, and a more obvious HfB_2_ diffraction peak. The formation of the HfSiO_4_ phase typically necessitates a higher temperature due to thermodynamic constraints. However, in this ablation experiment, the surface temperature of the sample was notably low. This may explain why no XRD peaks were observed. Furthermore, the XRD patterns of the surfaces of the HS to HS13 ceramic bulks after ablation were refined to calculate the relative percentage contents of HfB_2_ and HfO_2_. Figure 5F illustrates that the oxidation of HfB_2_ is more prevalent than that of SiC. However, after the oxidation of the HS to HS13 ceramic bulks, a substantial amount of the HfB_2_ phase remains on the surface. This indicates that the SiO_2_ phase, produced by the oxidation of SiC on the sample surface, is present in less quantities. A phase content that is too low can trigger error reports from the software during the fine finishing process; thus, it is not considered here (Fig. S9). The results show that after ablation, the surface of the HS ceramic bulk has the highest content of HfO_2_, about 91.9%. With the gradual increase in the addition amount of single-crystal HfB_2_ microrods, the HfO_2_ content on the surface of the samples after ablation shows a trend of gradual decrease. For the HS3 sample, the content of HfO_2_ is about 81.8%. When the addition of HfB_2_ microrods reached 13 wt.%, the HfO_2_ content of the ceramic bulk further decreased to 74.6%. This underscores the role of single-crystal HfB_2_ microrods in dissipating the temperature of the ceramic bulk surfaces during ablation, thus reducing the degree of oxidation of the surface of the ceramic bulks.

The plasma ablation process is oxygen enriched; therefore, the subsequent reactions (R1 to R10) may transpire during the ablation test [67–69]. The Gibbs free energy–temperature diagram associated with these reactions, extracted from the thermochemical data in the HSC software database, is illustrated in Fig. 5F. It can be deduced that reactions R1 to R4 are spontaneous under the experimental conditions, aligning with the findings of the XRD test results.

2HfB_2_ (s) + 5O_2_ (g) → 2HfO_2_ (s) + 2B_2_O_3_ (s)  R1
SiC (s) + 2O_2_ (g) → SiO_2_ (s) + CO_2_ (g)     R2
2SiC (s) + 3O_2_ (g) → 2SiO_2_ (s) + 2CO (g)   R3
SiC (s) + O_2_ (g) → SiO (g) + CO (g)      R4
SiC (s) + 2SiO_2_ (s) → 3SiO (g) + CO (g)    R5
SiC (s) + SiO_2_ (s) → 2SiO (g) + C (s)     R6
B_2_O_3_ (s) → B_2_O_3_ (l) → B_2_O_3_ (g)        R7
SiO_2_ (s) → SiO_2_ (l) → SiO_2_ (g)        R8
2C (s) + O_2_ (g) → 2CO (g)          R9
C (s) + O_2_ (g) → CO_2_ (g)           R10

**Fig. 6.**
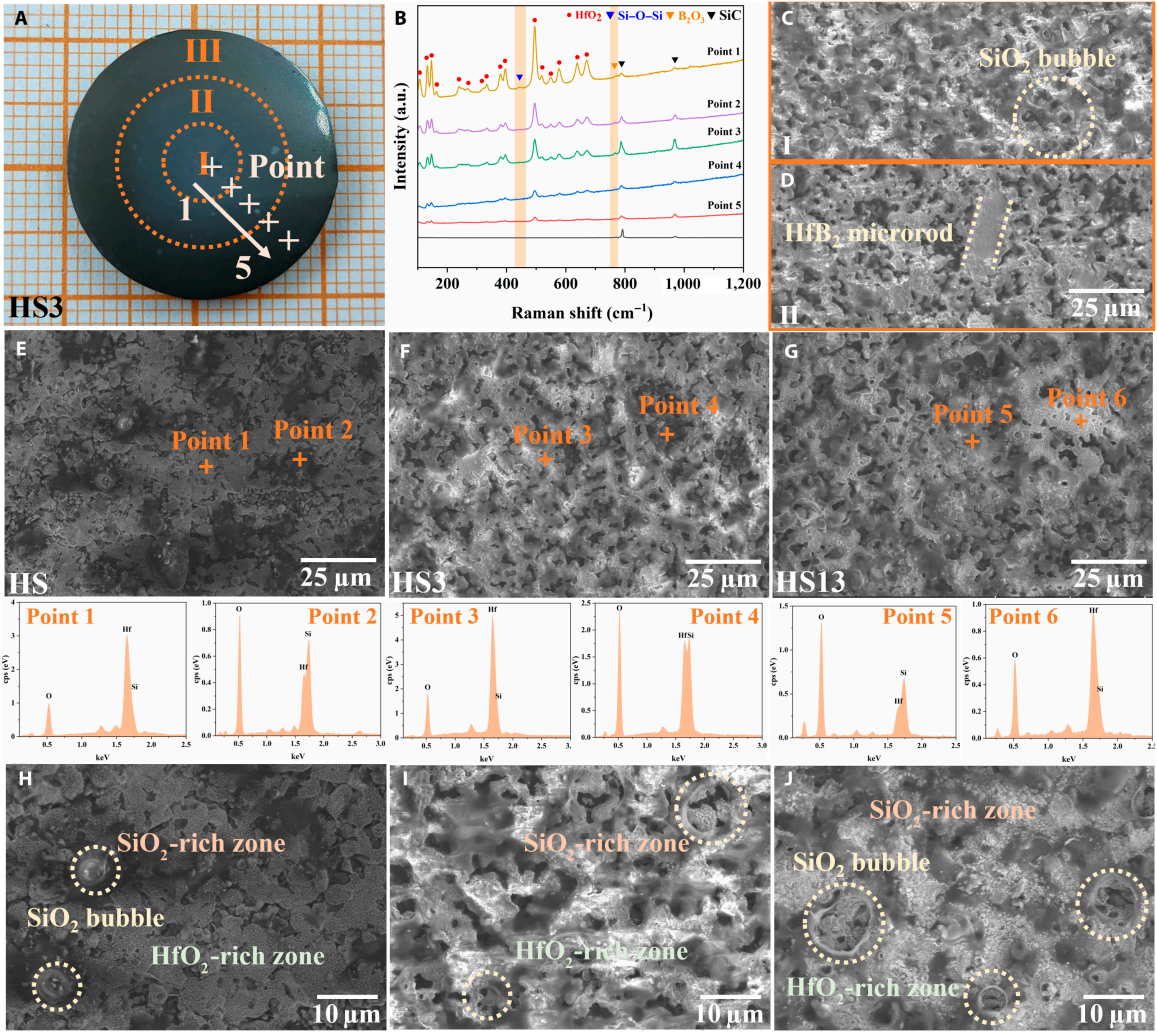
Optical image of the HS3 sample after ablation (A), Raman spectra selected from 5 points sequentially from the center area outward along the axis of the ceramic block (B), SEM image of ablation areas I and II of the HS3 ceramic bulk (C and D). Surface morphologies of the HS, HS3, and HS13 samples after ablation (E to J) and spot energy-dispersive spectroscopy (EDS) analyses.

Conducting a meticulous analysis of the microstructure is imperative for comprehending the superior ablation resistance exhibited by HfB_2_–SiC composites. Firstly, for the HS3 ceramic bulk, the surface of the ablated ceramic bulk is segmented into 3 distinct regions (I, II, and III) based on the distance from the ablation center. Five points were sequentially chosen along the axial direction (Fig. 6A), with the Raman spectra of these points measured as illustrated in Fig. 6B. The lowest black line in Fig. 6B represents the Raman spectrum of the HS3 ceramic bulk’s surface prior to ablation, where only the transverse optic peak of SiC at 796 cm^−1^ and the longitudinal optic peak at 972 cm^−1^ are observable in the spectrum [70]. At point 1, which is closest to the ablation center, there are pronounced peaks of HfO_2_ in the Raman spectrum, along with the stretching and bending vibration peaks of Si–O–Si at 416 cm^−1^ [71,72]. This suggests that an obvious oxidation reaction occurred at this position. However, the signal peak of B_2_O_3_ here is relatively weak, possibly due to the higher temperature during the ablation process, which results in more volatilization of B_2_O_3_. As one moves progressively away from the ablation center, the Raman peak intensity of HfO_2_ diminishes, while that of SiC increases, indicating a decrease in the degree of oxidation. When the measurement position is near the edge of the sample (point 5), only relatively weak peaks of HfO_2_ and SiC exist in the Raman spectrum, suggesting the lowest degree of oxidation here. The SEM images in Fig. 6C further corroborate this phenomenon. In region I, there are noticeable gray-black SiO_2_-rich areas and white HfO_2_-rich areas. In the SiO_2_-rich area, due to the escape of internal components during the ablation process, a large number of “SiO_2_ bubbles” form on the surface. The bursting of these bubbles accelerates the internal oxidation of the material. In region II, due to the reduced degree of oxidation, the “SiO_2_ bubbles” are visibly reduced, and one can observe the phenomenon where the HfB_2_ micro-rod grain boundaries are oxidized, while their centers remain unoxidized. In view of this, subsequent discussions on the micromorphology of the sample all select the ablation center.

Figure 6E to J show the SEM images of the surface near the ablation centers of HS, HS3, and HS13 ceramic blocks after ablation for 60 s. It is evident that these ceramic materials exhibit not entirely the same ablation morphology. Generally, the samples undergo mechanical erosion, thermal volatilization, and oxidation during the ablation process. The SEM images illustrated in Fig. 6E to J present the surface appearances of the samples following the ablation process. It is evident that a layer of molten SiO_2_ covers the surfaces. Additionally, the escape of SiO_2_ glass during ablation results in a discontinuous skeleton of HfO_2_ becoming discernible [67,73]. Overall, the surface microstructure of the post-ablated HfB_2_–SiC composites is primarily dense. EDS analysis conducted on points 3 to 6 reveals that the gray glass layer and white phase respectively correspond to Hf–Si–O glass and HfO_2_. For the HS ceramic bulk, the areas occupied by SiO_2_ exhibited a more random and less uniform distribution compared to those for the other samples; within the SiO_2_-rich regions on its surface, occasional “SiO_2_ bubbles” were still observed. In the HS13 ceramic bulk, the loss of SiO_2_ intensifies due to its low density, leading to an increased number of holes on the surface. The SiO_2_-rich region is more dispersed and contains a larger volume of SiO_2_ bubbles. When these bubbles rupture, they expose the interior of the HS13 ceramic block directly to the external environment, thereby accelerating its oxidation. During the oxidation process of the ceramic material, B_2_O_3_ provides exceptional protection in a liquid state below 1,100 °C. However, once the temperature surpasses 1,100 °C, B_2_O_3_ rapidly vaporizes due to its high vapor pressure (as detailed in reaction R7), and this process leaves a dense layer of HfO_2_ exposed to high temperatures. R2 shows that the addition of SiC facilitates the formation of a glassy SiO_2_ layer on the outermost surface, playing a crucial role in protecting the HfB_2_–SiC composites from oxidation up to 1,500 °C [67,73,74].

**Fig. 7.**
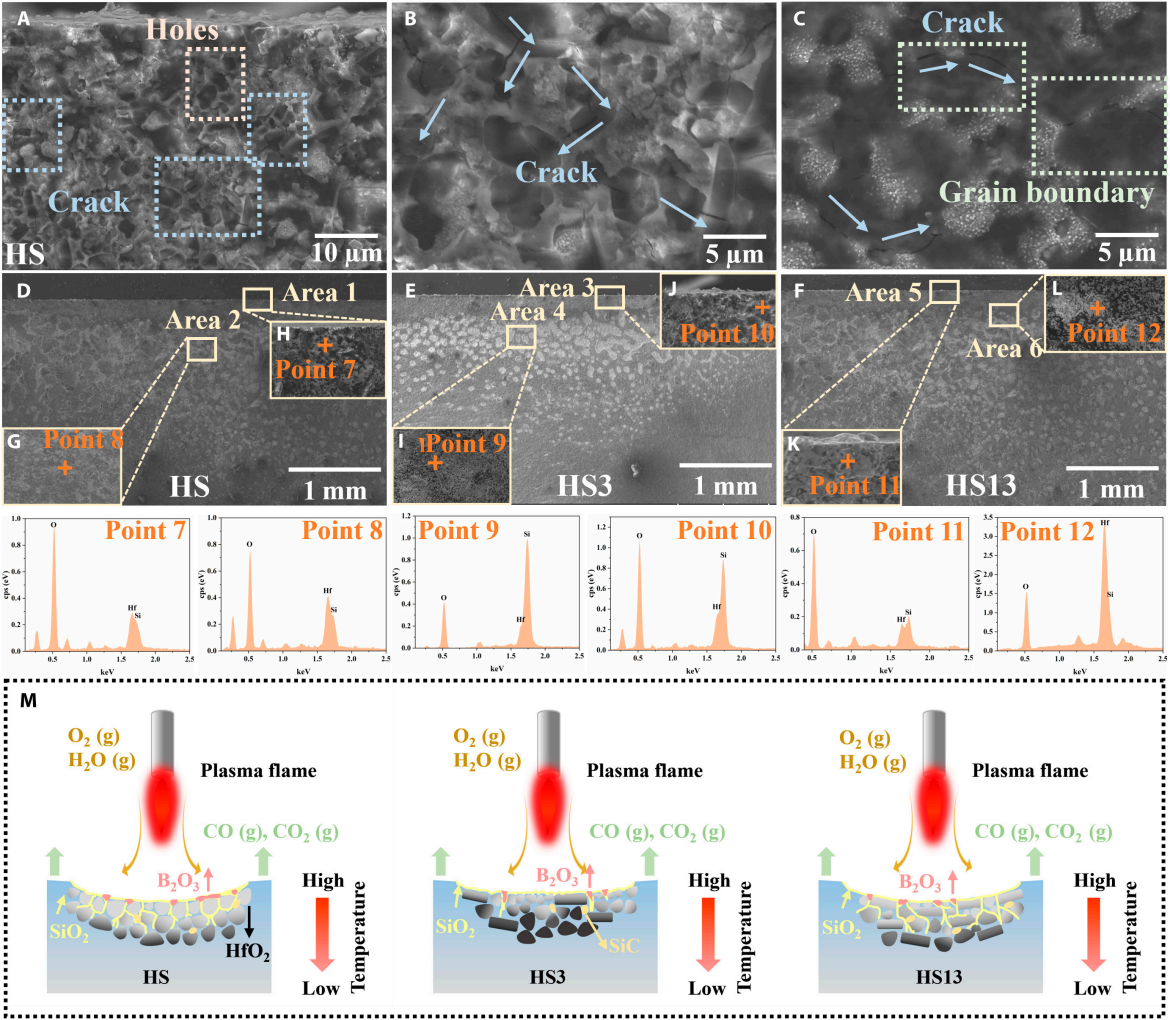
SEM images of the fracture surface of the HS ceramic bulk after ablation (A to C). Cross-sectional (D to L) morphologies of HS, HS3, and HS13 ceramic bulks after ablation and spot EDS analyses. Schematic diagram of the plasma flame ablation process (M).

To elucidate the impact of single-crystal HfB_2_ microrods on the ablation resistance of the material, Fig. 7A to F depict the microstructure of the samples at the ablation center cross-section. For TPMs, maintaining the integrity of the material at high temperatures is particularly important. Consequently, we chose the HS ceramic block exhibiting the most pronounced fragmentation post-ablation to conduct an in-depth analysis of the fracture surface proximate to the ablation center. The SEM image in Fig. 7A reveals an obvious presence of pores and microcracks on the fracture surface. This suggests that the disintegration of the ceramic material may initiate from these internal microcracks. The crack propagation path is more obvious at higher magnifications, and the cracks always extend along the direction of the grain boundary. It is speculated that during the oxidation process, the precipitated substances near the grain boundary gather to form bubbles, and then the bubbles connect with each other, and finally the internal thermal stress of the material will lead to the generation of cracks and the fragmentation of the material.

Figure 7D to L depict the microstructure at the regions near the surface and the inner areas of the samples. It is evident that the closer one gets to the ablation center, the deeper the oxygen element’s intrusion depth becomes. This appears as an inverted triangle distribution on the cross-section. However, for the HS ceramic bulk without the addition of single-crystal HfB_2_ microrods, the HfO_2_ phase is confined to a narrow range near the surface, but it has a greater distribution depth and tends to cluster. With the addition of 3 wt.% HfB_2_ microrods, the HfO_2_ phase extends over a wider range near the surface and is more horizontally dispersed, and its depth becomes shallower. After adding 13 wt.% HfB_2_ microrods, the HfO_2_ phase no longer follows an inverted triangle distribution pattern but diffuses horizontally. The HfO_2_ phase then becomes dispersed both horizontally and vertically. This phenomenon can be attributed to the higher thermal conductivity of single-crystal HfB_2_ microrods along the *c*-axis direction. Consequently, heat on the surface dissipates faster during the ablation process, leading to a more uniform surface oxidation. Specifically, the sample with 3 wt.% single-crystal HfB_2_ microrods displays a relatively shallower depth of oxidation erosion. Nevertheless, an excessive amount of rodlike HfB_2_ results in a decreased sample density, thereby failing to effectively impede the diffusion of oxygen elements within the material.

Combined with the results of first-principles calculations, it is found that its $\left(10\bar{1}0\right)$ crystal surface presents more difficulty when combining with O atoms than does the (0001) surface. Based on the CO maps of ceramic bulks, we speculate that single-crystal HfB_2_ microrods predominantly display a horizontal orientation within the material. Specifically, the $\langle 10\bar{1}0\rangle$ planes are more inclined to remain parallel to the sample surface, whereas the (0001) planes are preferred oriented perpendicularly. This orientation suggests that the $\langle 10\bar{1}0\rangle$ planes present a barrier when oxygen elements infiltrate the material’s interior. Consequently, oxygen must navigate around these planes for deeper penetration, thereby bolstering the material’s resistance to both oxidation and ablation. Overall, the integration of single-crystal HfB_2_ microrods serves a dual purpose. Firstly, the high thermal conductivity along the *c* axis expedites the heat transfer and dissipation on the sample surface throughout the ablation process. Secondly, by presenting a crystal surface with low reactivity, they effectively limit oxygen infiltration, thereby helping to improve the sample’s ablation resistance.

To summarize, a comprehensive schematic diagram illustrating the ablation process is presented in Fig. 7M. At the beginning of ablation, SiO_2_, a byproduct of SiC oxidation, migrates to the surface, culminating in the formation of a cohesive glassy layer. Concurrently, HfO_2_ is dispersed into this glassy matrix due to the segregation effects induced by the SiO_2_ flow. Differently, commercial polycrystalline HfB_2_ powder tends to oxidize almost simultaneously, while the addition of single-crystal HfB_2_ microrods delays the rate of oxygen element penetration, attributed to the low oxygen activity on its $\langle 10\bar{1}0\rangle$ crystal planes. This results in a more convoluted path for oxygen invasion. As the concentration of HfB_2_ microrods increases, there is a corresponding decrease in the density of the ceramic block. Consequently, the avenues for oxygen infiltration expand, diminishing the inhibitory effect of the single-crystal HfB_2_ microrods. Moreover, the resultant SiO_2_ exhibits low viscosity, rendering it susceptible to easy removal during flame brushing. SiO_2_ demonstrates a restricted ability to facilitate the healing of ablation defects and the sintering of HfO_2_. As ablation time prolongs, the SiC-depleted zone continually expands, forming a porous structure within the coating that enables rapid oxygen diffusion. This results in an increased oxidation rate of ceramic bulks and a progressive decline in protection capability.

## Conclusion

In conclusion, multiscale hierarchical architectured HfB_2_–SiC composites were successfully fabricated through SPS, exhibiting enhanced mechanical robustness and ablation resistance. The primary conclusions drawn from the study are summarized as follows: Compared with samples without added toughening phases, the composite material with added 6 wt.% single-crystal HfB_2_ microrods demonstrated increases in hardness and fracture toughness of 4.1% and 37.6%, respectively. First-principles calculations indicate that compared to the (0001) crystal plane, the $\langle 10\bar{1}0\rangle$ crystal planes exhibit a reduced reaction activity. Further, electron backscatter diffraction results suggest that the $\langle 10\bar{1}0\rangle$ crystal planes of the single-crystal HfB_2_ microrods are more inclined to align parallel to the sample surface. This orientation impedes the ingress of oxygen atoms upon penetration, thereby helping to bolster the material’s oxidation and ablation resistance. In conjunction with the plasma flame ablation experiment, it has been confirmed that the composite containing 3 wt.% single-crystal HfB_2_ microrods demonstrates superior ablative characteristics. The mass ablation rate is measured at −0.013 mg/s, while the linear ablation stands at 0.25 μm/s. In totality, single-crystal HfB_2_ microrods, with their high aspect ratio, promote the deflection and bridging of cracks in ceramic materials, subsequently augmenting their hardness and fracture toughness. Concurrently, the minimal reactivity of their exposed $\langle 10\bar{1}0\rangle$ crystal planes establish a basis for enhanced oxidation resistance. This work offers a new train of thought and reliable solutions for addressing the brittleness and insufficient ablation resistance of traditional UHTCs. Future exploration should focus on optimizing the ceramic microrod components and material design to prepare novel hybrid reinforced structures.

## Materials and Methods

### Materials

Commercial HfB_2_ powders (*D*_50_ = 0.5 to 1 μm) and SiC powders (*D*_50_ = 0.5 to 0.7 μm) were purchased from Forsman. The multibranched HfB_2_ powders were self-made [29] (the SEM image and aspect ratio distribution of the powders are shown in Fig. S10). Ethanol absolute was supplied by Sinopharm Chemical Reagent Co. Ltd, China. Deionized water, with a resistivity of 18.20 MΩ·cm, was employed throughout the duration of the experiment (the mixing details of the raw powders are described in Part S1 of the Supplementary Materials).

### Preparation of the ceramic bulks

The process of fabricating ceramic bulks is illustrated in Fig. S11. Initially, the mixed ceramic powders were prepared by blending different mass ratios of single-crystal HfB_2_ microrods with commercial HfB_2_ powders. These compositions included the original commercial HfB_2_ and SiC powders, with subsequent additions of HfB_2_ microrods at 3, 6, 10, and 13 wt.%, which were denoted as HS3, HS6, HS10, and HS13, respectively. To ensure a homogeneous mixture, the combined powders were ultrasonicated to dispel agglomerates, followed by a 5-h stirring period in absolute ethanol. After thorough drying, the mixtures were placed in a graphite die (20-mm diameter) and sintered using an SPS system (LABOX-350, Sumitomo, Japan) in an argon atmosphere (see Part S1 of the Supplementary Materials for details).

### Bulk characterizations

Employing Archimedes’ method, the bulk densities (*ρ_bulk_*) of the ceramic bulk were measured. The relative densities (*ρ_relative_*) were subsequently derived by comparing these measured bulk densities to the theoretical density.

The mechanical properties, namely, room-temperature Vickers hardness (*H_v_*) and fracture toughness (*K_IC_*), were assessed by averaging 15 measurements taken with a Vickers hardness tester (VH1202, Wilson, America). The tests were conducted under an indenter load of 4.90 N, with a loading duration of 15 s. The fracture toughness was then calculated using the equation given as [75]

$$K_{\mathrm{IC}}=0.16H_{V}\times a^{2}\times c^{-\frac{3}{2}}$$ (4)

Nanoindentation tests were performed on sintered pellets using a nanoindenter (KLA iMicro, USA) under a 500-μN applied load and a Berkovich tip. The Oliver–Pharr method was utilized for calculating the elastic modulus. Each specimen underwent 5 indentations spaced 20 μm apart to avoid interaction effects.

To measure the elastic modulus of the ceramic blocks, a cube of dimensions 2 × 3 × 2 mm was cut from the H0 to HS13 ceramic bulks using a diamond wire cutting machine. Subsequently, an Instron-5967 electronic material testing machine was employed to compress the H0 to HS13 ceramic bulks at a steady rate of 0.06 mm/min, corresponding to a strain rate of 2 × 10^−4^ s^−1^. Each sample was tested at least 3 times.

The phase composition of ceramic bulks was characterized by XRD (X’Pert Pro, PANalytical, the Netherlands; Cu Kα, *λ* = 1.54 Å). The XRD data refinement was accomplished by the GSAS software. Microstructural morphology and elemental distribution were analyzed using SEM (SU8020, Hitachi, Japan) with EDS.

For confocal microprobe Raman measurements (Renishaw inVia Reflex, England), excitation light with a wavelength of 532 nm was vertically projected onto the sample and the effective power of the laser source was 10 mW.

### Computational details

Calculations utilizing density functional theory were conducted employing the Vienna Ab initio Simulation Package program, which is founded on a plane wave basis set. The studies presented in this paper were all executed at the spin-polarized generalized gradient correction level of density functional theory. For these calculations, the Perdew−Burke−Ernzerhof functional was used. For HfB_2_ slabs, a cutoff energy of 450 eV and Monkhorst–Pack grid *k* points of 8 × 8 × 1 were utilized, in accordance with the convergence test method detailed in previous studies. The convergence criterion was 10^−6^ eV for self-consistent electronic minimization. Structural optimization was stopped when the *x*, *y*, and *z* components of the atomic forces were smaller than 0.05 eV/Å. The determined lattice constants for HfB_2_, namely, *a* = 3.154 Å and *c* = 3.501 Å, align well with both experimental findings (*a* = 3.14 Å and *c* = 3.48 Å) and other theoretical predictions. Moreover, the calculated volume and formation enthalpy of HfB_2_ were 30.16 Å^3^ and −3.05 eV, respectively, which also closely align with both other computed values and experiment results [51,52,76,77] (further calculation details are provided in Supplementary Materials Part S1).

### Oxidation tests

#### Nonisothermal oxidation tests

Small specimens of H0 to HS13, measuring approximately 2.0 × 3.0 × 1.0 mm and weighing about 50 mg, were placed in alumina crucibles. These were then heated at a rate of 10 °C/min from 30 to 1,500 °C in a thermogravimetric analyzer (TGA/DSC 3+, METTLER TOLEDO, USA) under a flow of air. The mass change of the specimens during heating was recorded, using dry alumina (Al_2_O_3_) powders as reference. Each sample was tested 3 times to eliminate the influence of random errors.

#### Isothermal static oxidation tests

The static-isothermal oxidation test was conducted at 1,500 °C using a high-temperature furnace. Three samples were placed in the chamber at the same time for testing to eliminate the influences of random error. Prior to the isothermal oxidation test, the initial mass (*m*_0_) and the total surface area (*S*) of each sample were measured and recorded. Following exposure to the target oxidation temperature for a predetermined period, samples were removed from the furnace and allowed to cool to room temperature in air. Subsequently, they were weighed (*m_t_*) to compute the mass change unit area (Δ*M_S_*) by the following equation [67]:

$$\Delta M_{S}\%=\frac{m_{t}-m_{0}}{S}\times 100\%$$ (5)

### Ablation tests

Plasma ablation tests, compared to oxidation experiments, provide a more rigorous assessment of a block’s performance under extreme conditions, simulating a harsher service environment. The ablation tests were carried out using a self-developed bench based on a plasma generator (Multiplaz 3500). The temperature of the plasma flame was maintained at 2,000 °C (2,273 K), with an ablation duration of 60 s. The linear ablation rates were calculated by assessing the change in the thickness at the center of the sample before and after ablation, using Eq. (6). Furthermore, the mass ablation rates of the specimens were quantified using Eq. (7) [69,74].

$$R_{l}=\frac{\Delta d}{t}=\frac{d_{1}-d_{2}}{t}$$ (6)

$$R_{m}=\frac{\Delta m}{t}=\frac{m_{1}-m_{2}}{t}$$ (7)

The formulae mentioned above can be expressed as follows: *R_l_* represents the linear ablation rate; *d*_1_ and *d*_2_ denote the thickness at the top of the hemisphere before and after ablation, respectively. To ensure accuracy, the thickness of the samples was measured at 10 different points near the center, both pre- and post-ablation. The average value of these 10 measurements was then determined; *R_m_* signifies the mass ablation rate; *m*_1_ and *m*_2_ are the sample masses before and after ablation, respectively; and *t* is the ablation time. The ultimate ablation value is derived from averaging the measurements of 3 distinct specimens (see Part S1 of the Supplementary Materials for details).

## Data Availability

Data generated during the current study are available from the corresponding authors upon reasonable request.

## Supplementary Materials

Supplementary 1 Part S1: Experimental Details Part S2: The Process of Formula Derivation Figs. S1 to S11 Tables S1 to S3
