# Supplementary material for: Single-Crystal HfB2 Nanorod-Induced Synergy in HfB2–SiC Ultrahigh-Temperature Ceramics: Enhancement of Mechanical and Ablation Resistance
Source: Research (Wash D C). 2025 Nov 6;8:0963. doi: 10.34133/research.0963 (PMC12589770; doi:10.34133/research.0963)
Supplement: Supplementary 1 — Part S1: Experimental Details Part S2: The Process of Formula Derivation Figs. S1 to S11 Tables S1 to S3 [file research.0963.f1.docx]

Single-Crystal HfB_2_ Nanorod-Induced Synergy in HfB_2_-SiC UHTCs: Enhancement of Mechanical and Ablation Resistance

Kewei Li^1,2^, Zhen Wang^*,1^, Mulan Yu^1^, Mengen Hu^1,2^, Zhulin Huang^1,2^, Yuan Cheng^*,1,3^, Xiaoye Hu^*,1,2^, Yue Li^1,4^, Ping Hu^1,3^, Xinghong Zhang^1,3^

1 Key Laboratory of Materials Physics and Anhui Key Laboratory of Nanomaterials and Nanotechnology, Institute of Solid State Physics, HFIPS, Chinese Academy of Sciences, Hefei, 230031, China

2 University of Science and Technology of China, Hefei, 230026, China

3 Harbin Institute of Technology, Harbin, 150001, China

4 Tiangong University, Tianjin, 300387, China

**Part 1: Experimental details**

The mixing details of raw material powders

Firstly, the multi-branched HfB_2_ powders were subjected to 2 hours ultrasonic treatment to ensure complete dispersion, after which they were combined with commercial powders. The mixture was subjected to further ultrasonic treatment and stirred for 5 hours. The mixed paste was subjected to a drying process at 40 °C, followed by thorough oscillation using a constant temperature oscillator for approximately 10 hours.

SPS details

The powders were wrapped by a 0.50 mm-thick graphite paper to prevent adhesion between the specimen and the graphite mold. A double layer of graphite blanket was placed around graphite die, minimizing the heat loss. Temperature monitoring during the process is facilitated by an optical pyrometer (IR-AHS2, Chino, Japan), gauging the temperature at a depth of 2.50 mm within the graphite die. The sintering cycle was adopted as follows: increasing temperature up to 1600 ℃ with heating rate of 100 ℃/min and holding for 10 min, then ramping temperature up to 1800 ℃ and holding for another 10 min. A uniaxial pressure 30 MPa was applied throughout the sintering cycle.

Computational details

To clarify the interaction information between HfB_2_ surface and oxygen, the (0001), (11$\bar{2}$0), and (10$\bar{1}$0) surfaces of HfB_2_ were selected considering that the XRD diffraction peaks of the (0001), (11$\bar{2}$0) and (10$\bar{1}$0) planes are more pronounced than other planes. In order to distinguish the slab with different terminated atoms, the naming convention is as follows: (0001) slab with Hf and B atom terminations were labeled as: Hf-(0001) and B-(0001), respectively. (10$\bar{1}$0) slab with different first and second-layer terminating atoms are labeled as: Hf-B-(10$\bar{1}$0), B-Hf-(10$\bar{1}$0), and B-B-(10$\bar{1}$0), respectively, where the first and second elements represent the first and second-layer atom of HfB_2_(10$\bar{1}$0) slab, (11$\bar{2}$0) slab was stoichiometric structure, as presented in Fig. 4.

The convergence tests proved that a 11-layer Hf-(0001), 11-layer B-(0001), 11-layer Hf-B-(10$\bar{1}$0), 13-layer B-B-(10$\bar{1}$0), 13-layer B-Hf-(10$\bar{1}$0), and 12-layer (11$\bar{2}$0) slab with a vacuum layer of 20 Å were sufficient to avoid interaction between surfaces of neighboring slabs, and yielded well-converged results. the surface supercell was extended to 3 × 3.

Ablation tests details

The plasma (operating current of 6 A, voltage of 160 V with H_2_O) and compressed air flow (pressure of 0.4 MPa, flow rate of 2 m^3^/h), along with the inner diameters of both the plasma and airflow nozzles were 2 mm. During the ablation process, the surface center temperature of the ablated specimen was monitored by two infrared thermometers: the Endurance E3ML (323−1273 K, accuracy ± 0.3%, response time 20 ms) and the E1RH (1273−3473 K, accuracy ± 0.5%, response time 10 ms).

**Part 2: The process of formula derivation**

As for the (0001) and (10$\bar{1}$0) surfaces with a non-stoichiometric structure, Hf: B $\neq$ 1: 2, the chemical potential of Hf and B atoms must be considered, and the surface energy can be given as follows:

$\gamma_{surf}=\left(E_{slab}-nE_{bulk}\right)/2A_{s}$ (1)

Where $\mu_{Hf}^{slab}$and $\mu_{B}^{slab}$is the chemical potential of Hf and B atom, respectively.

For the bulk materials, its chemical potential is equal to the sum of the chemical potentials of each element in the slab, so the following equation exists:

(2)

The surface energy of a non-stoichiometric structure can be articulated as:

$\gamma=\frac{1}{2A}\left[E_{HfB_{2}}^{slab}-N_{Hf}\times\mu_{HfB_{2}}^{bulk}+\left(2N_{Hf}-M_{B}\right)\times\mu_{B}^{slab}\right]$ (3)

In addition, the elemental chemical potential of the bulk is also related to the enthalpy of formation of the bulk materials:

$\mu_{Hf}^{bulk}+2\mu_{B}^{bulk}+\Delta H_{HfB_{2}}=\mu_{HfB_{2}}^{bulk}$ (4)

Combining Eq. (2) with Eq. (4) yields the following equation:

$\mu_{Hf}^{bulk}+2\mu_{B}^{bulk}+\Delta H_{HfB_{2}}=\mu_{Hf}^{slab}+2\mu_{B}^{slab}$ (5)

After simple derivation calculations, the following inequality can be obtained:

$\frac{1}{2}\Delta H_{HfB_{2}}<\mu_{B}^{slab}-\mu_{B}^{bulk}<0$ (6)

**Part 3: Figures and Tables**


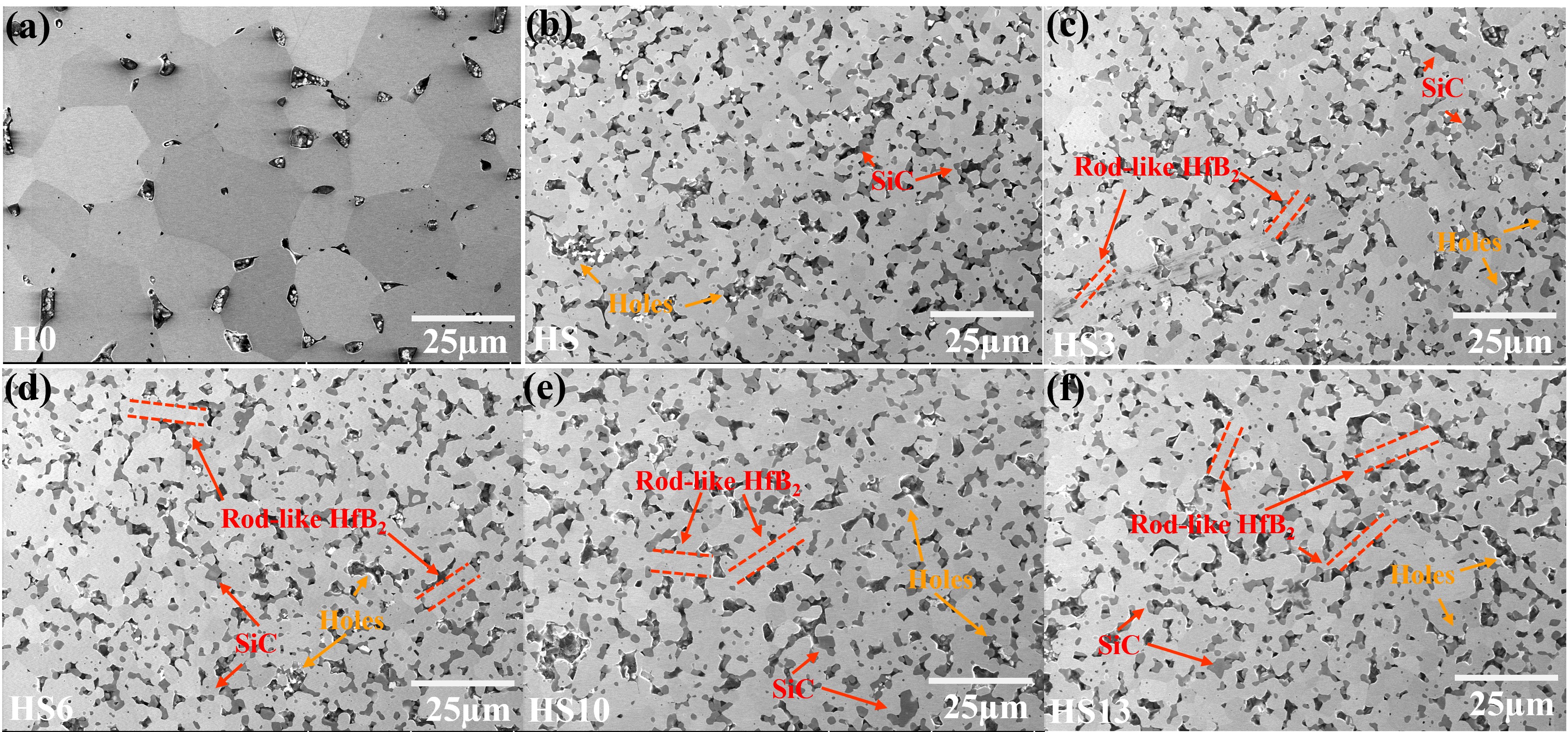


Fig. S1. SEM images of the surface morphology of H0-HS13 ceramic bulks.


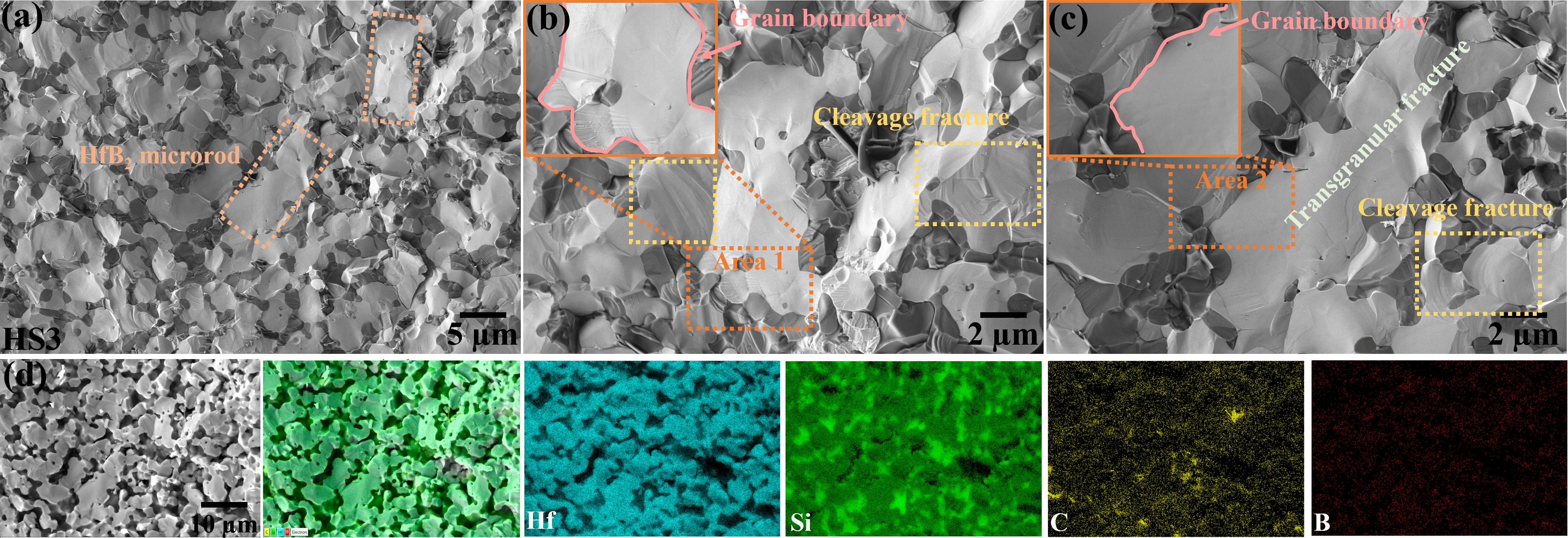


Fig. S2. Fracture surface morphology of HS3 ceramic bulk (a), microscopic morphology of HfB_2_ microrod region (b, c) and EDS mapping (d) on fracture surface.


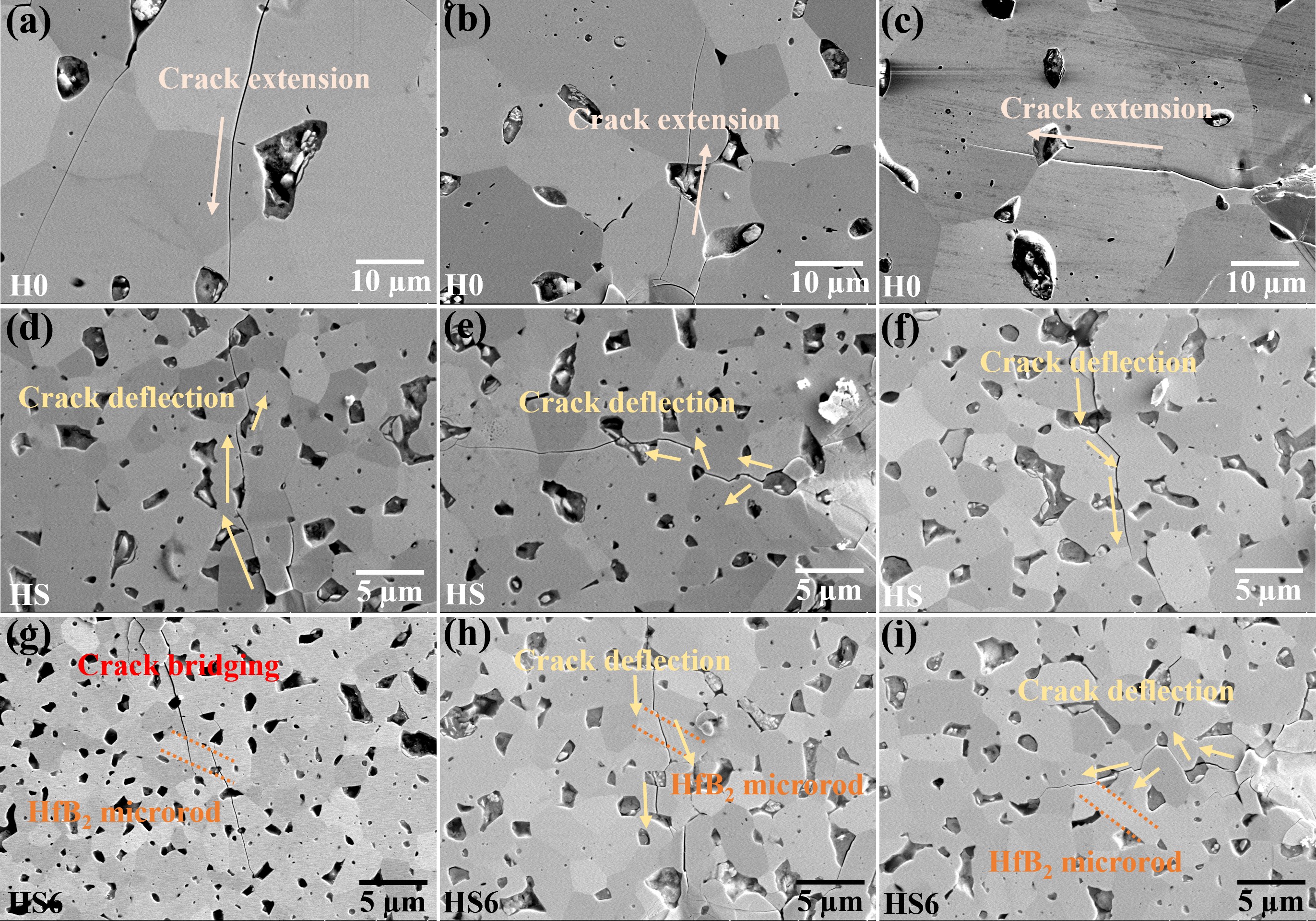


Fig. S3. Surface indentation morphology of H0 (a-c), HS (d-f) and HS6 (g-i) ceramic bulks.


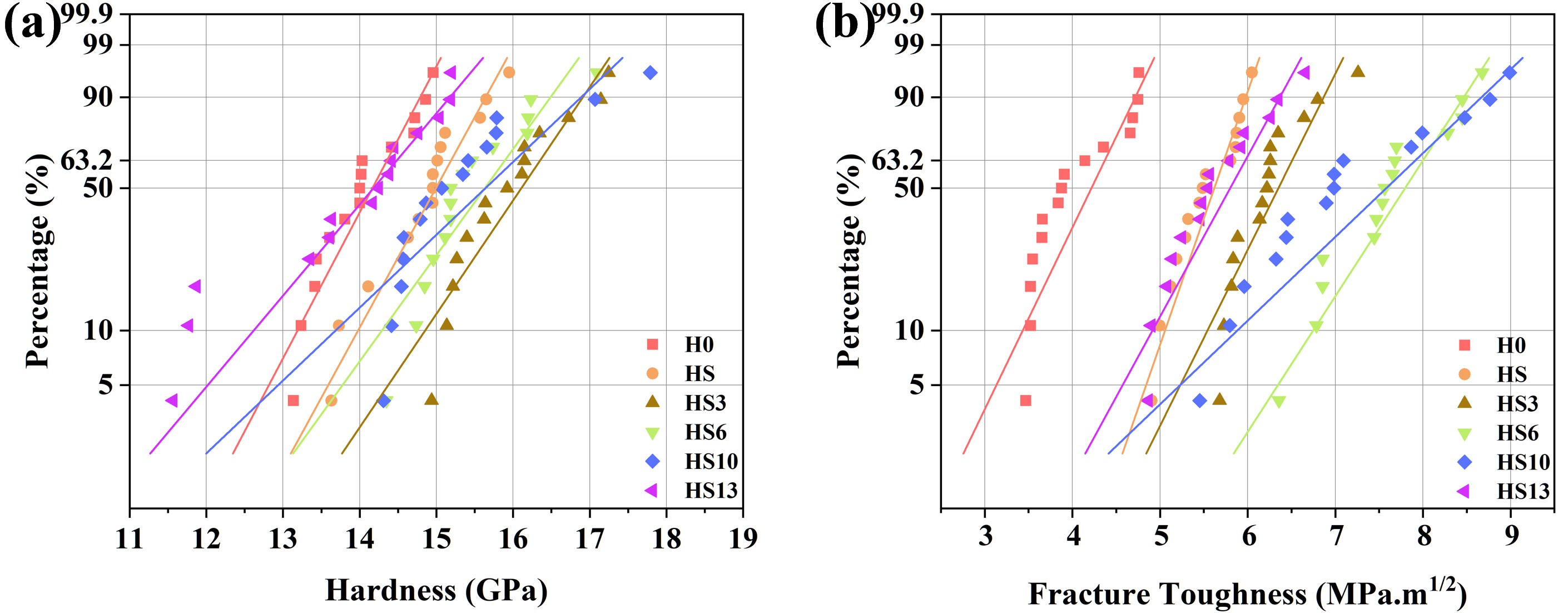


Fig. S4. Weibull distribution diagrams of surface hardness (a) and fracture toughness (b) data of H0-HS13 ceramic bulks.


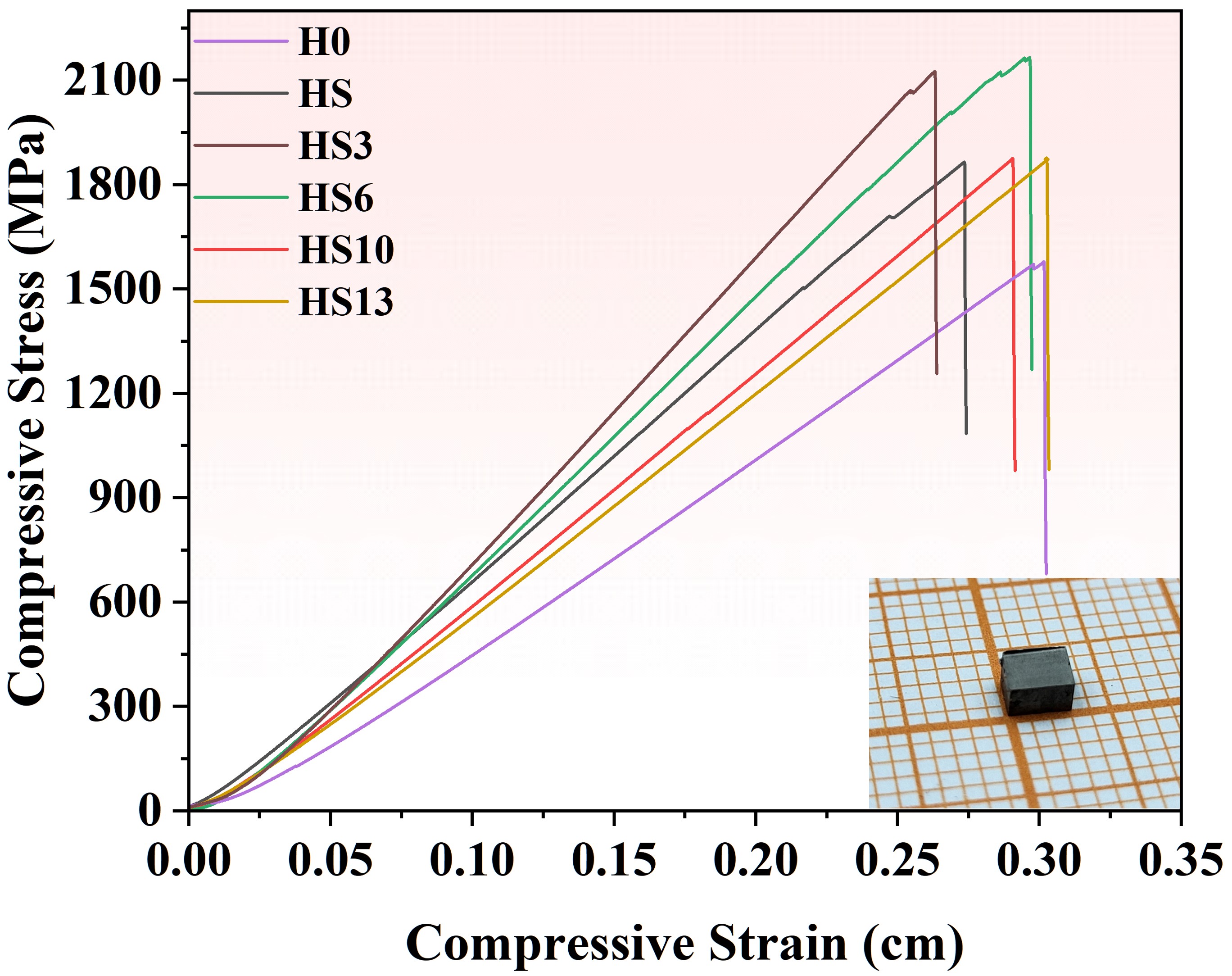


Fig, S5. Compressive stress of H0-HS13 ceramic bulks and optical images of test sample.


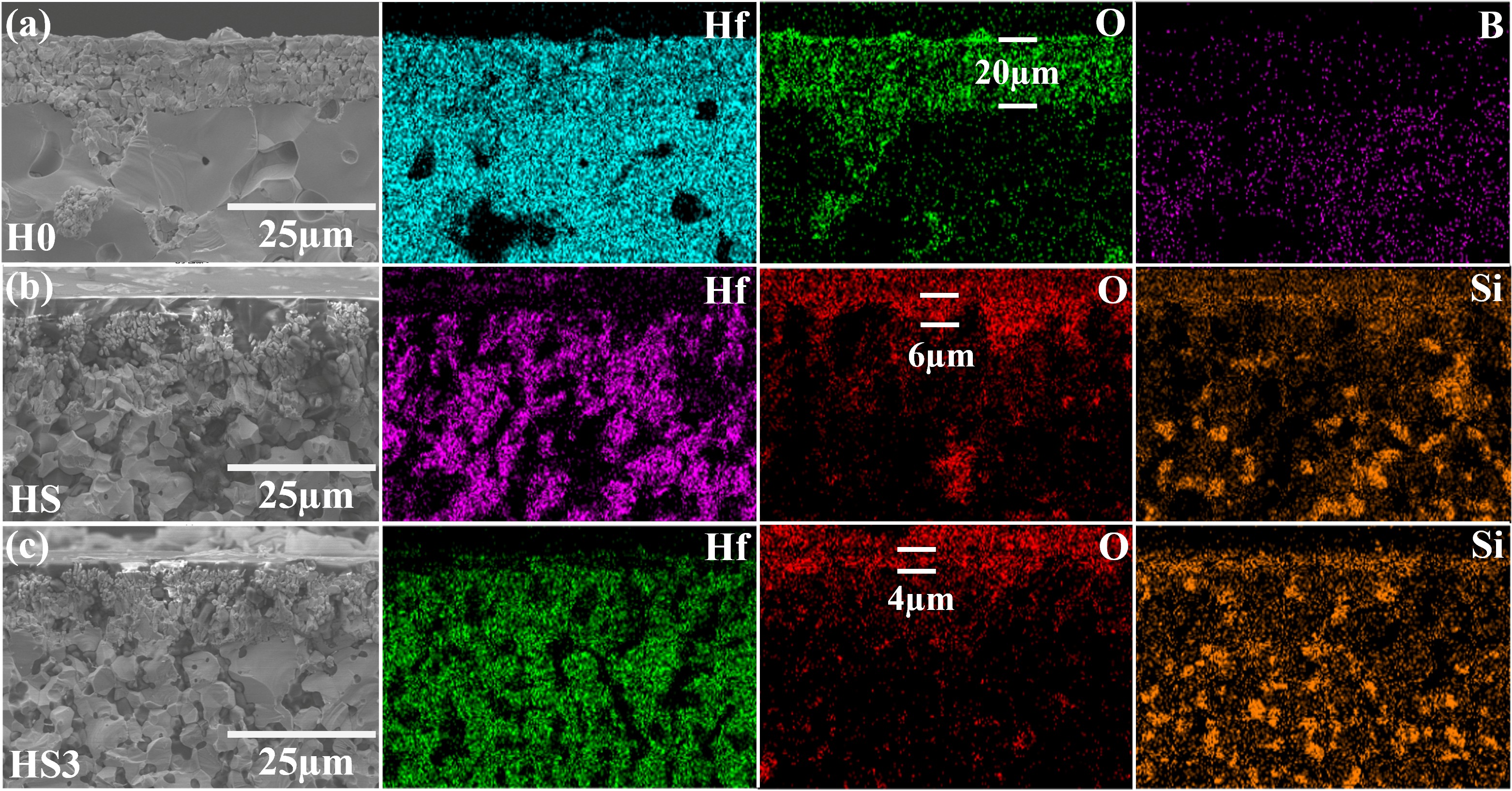


Fig. S6. EDS mapping of H0 (a), HS (b) and HS3 (c) bulks after oxidation on the cross-section.


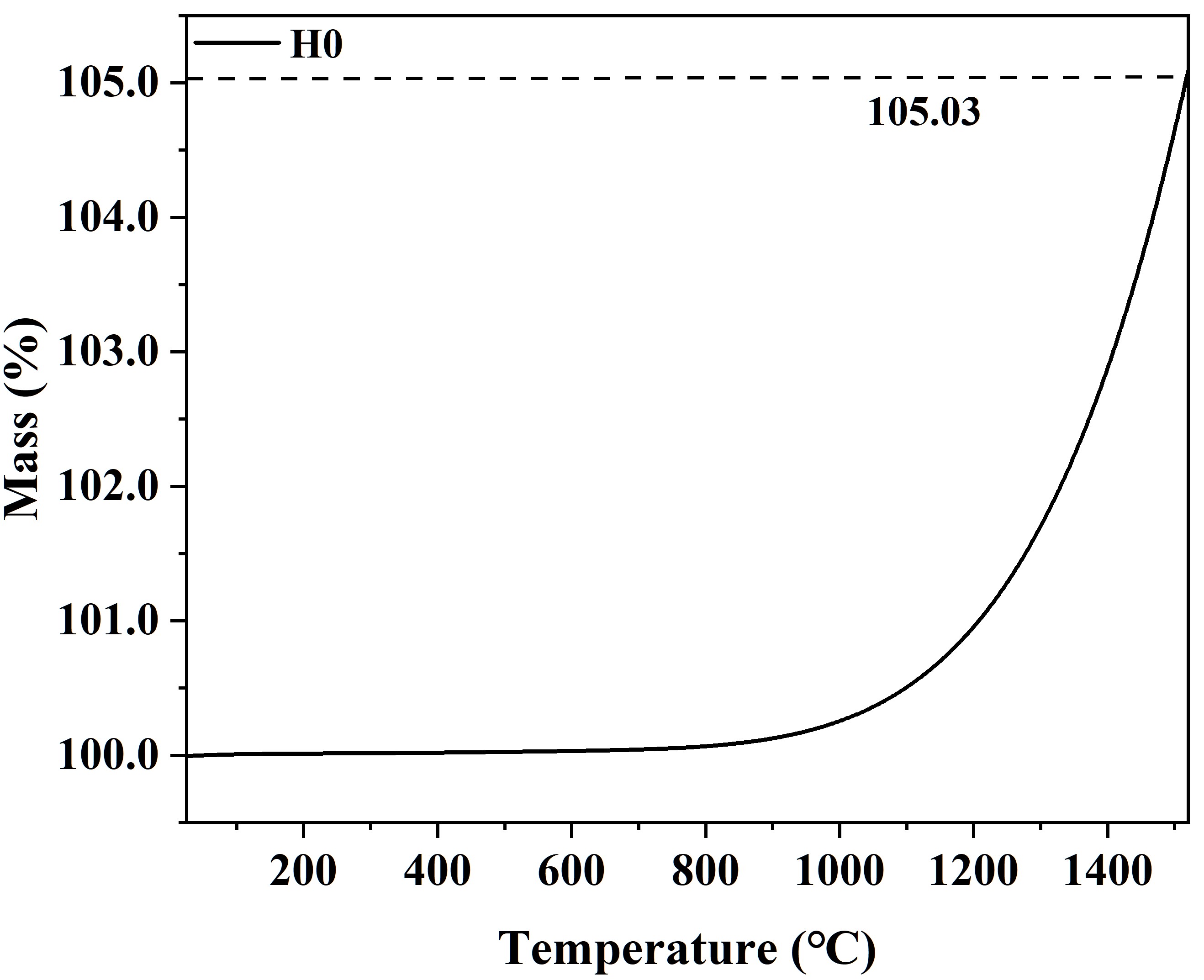


Fig. S7. Weight gain in the non-isothermal oxidation of H0 ceramic bulk.


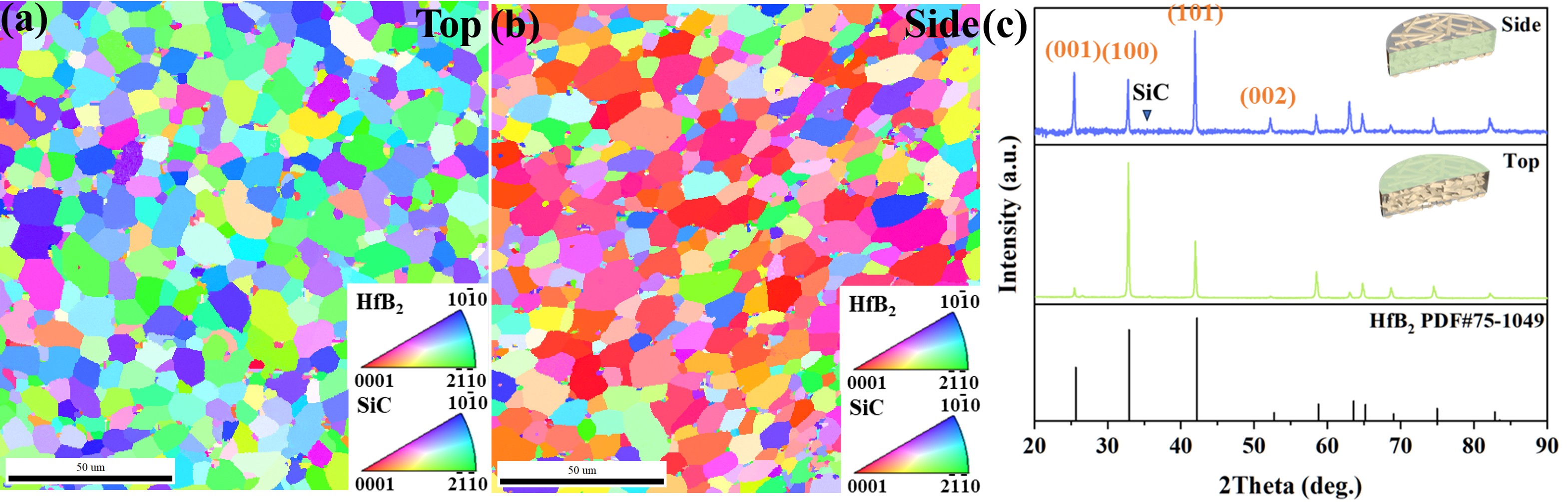


Fig. S8. CO map of the ceramic bulks prepared by HfB_2_ microrods and SiC on the top surface (a) and side surface (b), as well as XRD patterns of the top surface and side surface (c) of ceramic bulk.


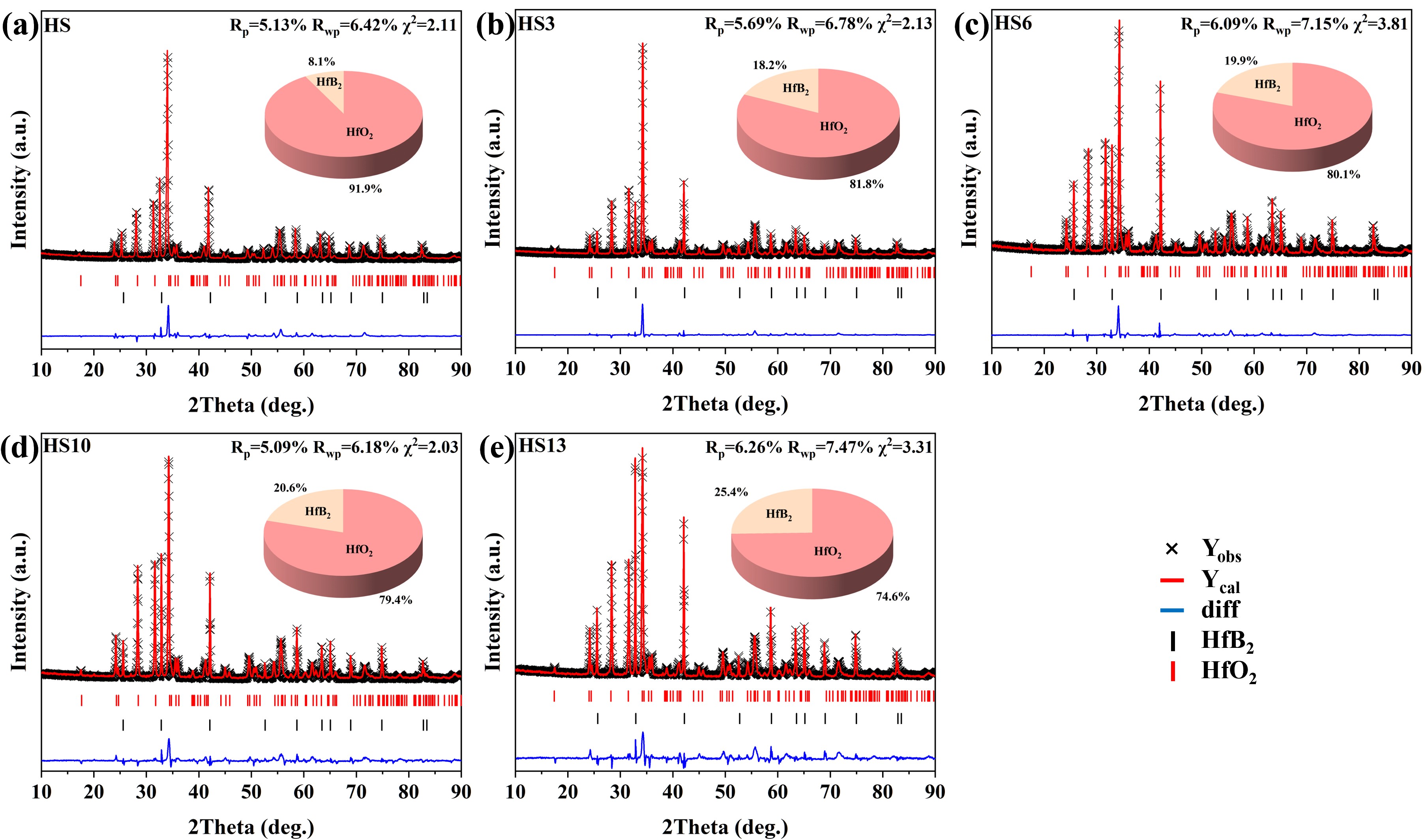


Fig. S9. Rietveld refined XRD patterns of HS-HS13 ceramic bulks after ablation.


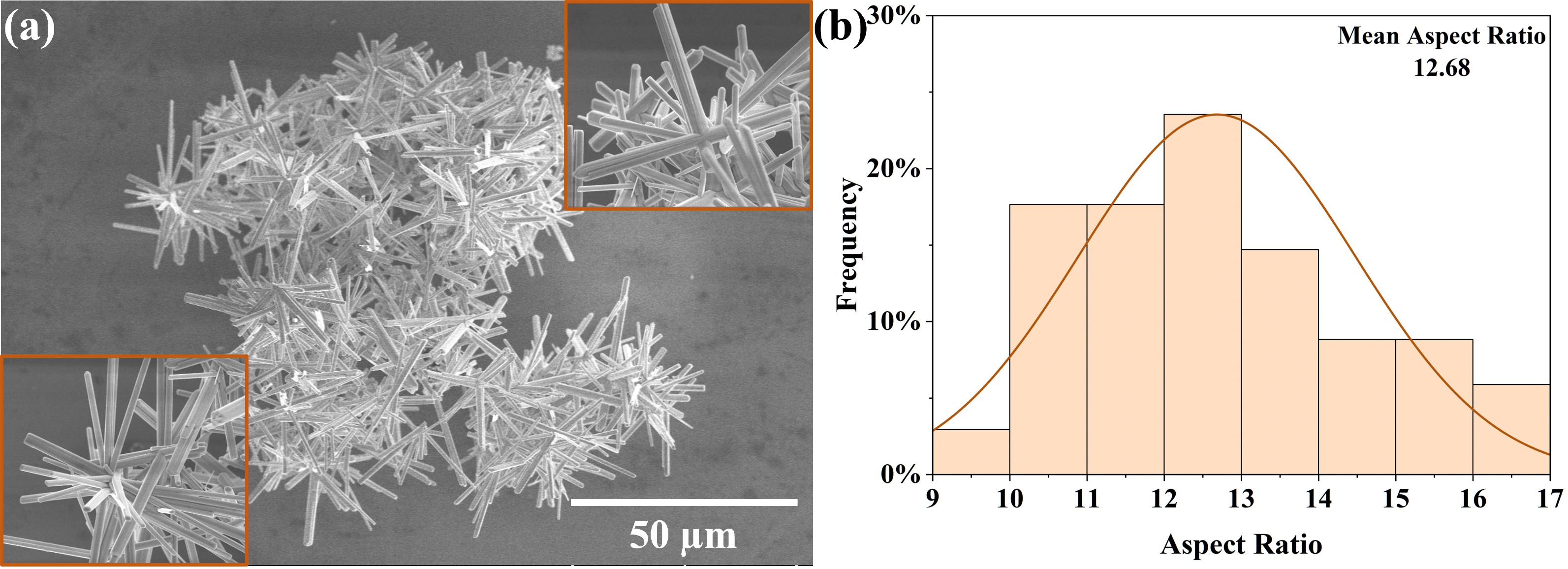


Fig. S10. Overall morphology and regional magnified images of multi-branched HfB_2_ powders (a), as well as the aspect ratio distribution (b).


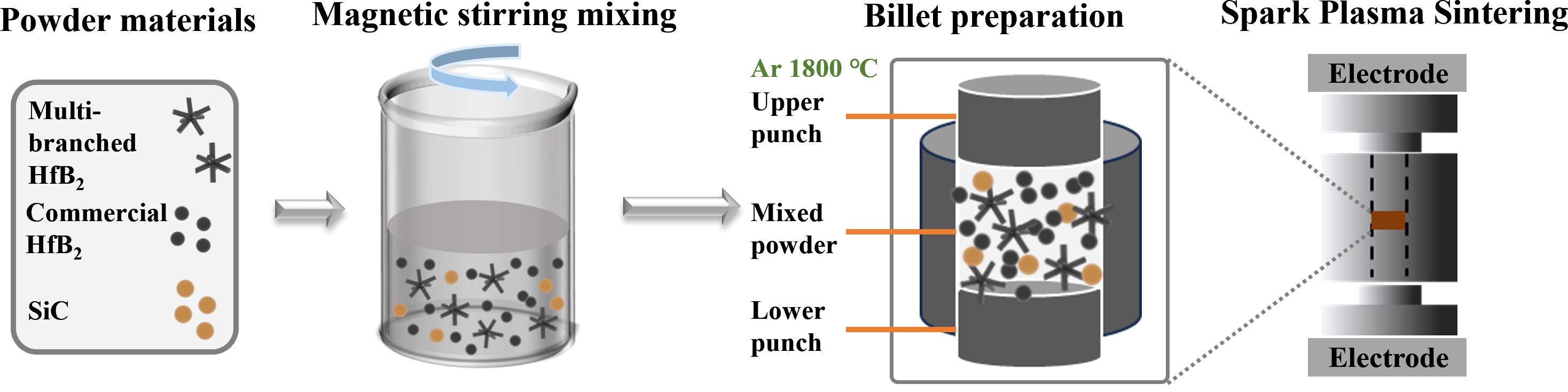


Fig. S11. Schematic diagram of the SPS process to compact mixed powders into bulks.

| **Material Composition** | **Sintering condition** | **Hardness (GPa)** | **Fracture Toughness**  **(MPa.m^1/2^)** |
| --- | --- | --- | --- |
| HfB_2_-20vol%SiC | SPS/1800℃/40MPa | 15.95 | 6.43 |
| HfB_2_-20vol%SiC | HP/2000℃/30MPa | 21.07 | 3.72 |
| HfB_2_-20vol%HfC-20vol%SiC | HP/2000℃/30MPa | 21.46 | 5.05 |
| HfB_2_-20vol%SiC | HP/2000℃/30MPa | 21.10 | 3.95 |
| HfB_2_-10vol%HfC-10vol%SiC | HP/2000℃/30MPa | 20.17 | 3.36 |
| HfB_2_-20vol%SiC | HP/1950℃/30MPa | 23.00 | 4.10 |
| HfB_2_-30vol%β-SiC | SPS/2100℃/30MPa | 26.00 | 3.90 |
| HfB_2_-19vol%SiC-Si_3_N_4_ | HP/1850℃/30MPa | 20.40 | 4.30 |
| HfB_2_-10vol%SiC-20vol%VSi | PS/2150℃ | 20.10 | 5.80 |
| HfB_2_-20vol%SiC-5vol%WC | HP/2000℃/30MPa | 22.30 | 3.76 |
| HfB_2_-SiC-MoSi_2_ | RSPS/1850℃/40MPa | 25.20 | 5.10 |
| HfB_2_-20vol%SiC | PS/2050℃ | 19.10 | 4.90 |
| HfB_2_-20vol%SiC  (HS3, This Work) | SPS/1800℃/30MPa | 15.45 | 7.58 |

Tab. S1. Comparison of the mechanical properties of the materials in this work with similar material systems.

| Sample | Compressive stress (MPa) | Elastic modulus (GPa) |
| --- | --- | --- |
| H0 | 1558.71 | 562.31 |
| HS | 1856.22 | 685.46 |
| HS3 | 2125.02 | 787.64 |
| HS6 | 2165.56 | 703.82 |
| HS10 | 1864.44 | 680.03 |
| HS13 | 1866.94 | 665.31 |

Tab. S2. Compressive stress and elastic modulus of H0-HS13 ceramic bulks.

| **Material Composition** | **Flame Temperature (℃)/Time (s)** | **Mass Ablation Rate (mg/s)** | **Linear Ablation Rate (μm/s)** |
| --- | --- | --- | --- |
| SiC-ZrB_2_-SiC | 1900/20 | -0.96 | -0.50 |
| ZrB_2_-SiC Coating | 2000/60 | 0.27 | 4.48 |
| ZrB_2_-SiC Coating-La_2_O_3_ | 2000/60 | 0.13 | 2.45 |
| ZrB_2_-SiC Coating | 1726/40 | 0.062 | 4.4 |
| ZrB_2_-SiC | 1900/120 | 0.576 | 1.5 |
| HfB_2_-SiC (this work) | 2000/60 | -0.013 | 0.25 |
| ZrB_2_-SiC-AlN | 2000/300 | 0.70 | 0.10 |
| ZrB_2_-SiC | 3000/300 | -0.11 | -0.30 |
| MoAlB | 2000/300 | -0.767 | -3.63 |
| MoAlB | 2000/600 | -0.623 | 1.71 |
| Ti_3_AlC_2_ | 1900/120 | 8.7 | 2.103 |
| ZrB_2_-SiC-ZrC Coating | 2000/120 | -0.016 | 1.3 |
| (TiZrHfNbTa)C | 2000/120 | 3.8 | -3.6 |
| (TiZrHfNbTa)C-SiC | 2000/120 | -1.9 | -2.1 |

Tab. S3. Comparison of the ablation resistance of the materials in this work with other UHTCs reported in the reference under similar flame temperatures.
